# P7C3-A20 Attenuates Microglial Inflammation and Brain Injury after ICH through Activating the NAD^+^/Sirt3 Pathway

**DOI:** 10.1155/2023/7857760

**Published:** 2023-02-08

**Authors:** Yang Wu, Qing Hu, Xun Wu, Ya-ning Cai, Yun-ze Zhang, Ying-xi Wu, Gang Zhu, Jia-ning Luo, Hong-bo Cheng, Jie-gang Yu, Xiao-liang Wang, Li Gao, Guo-dong Gao

**Affiliations:** ^1^Department of Neurosurgery, Tangdu Hospital, Fourth Military Medical University, Xi'an, Shaanxi Province 710038, China; ^2^Department of Neurosurgery, The Second Hospital of Hebei Medical University, Shijiazhuang, Hebei Province 050000, China; ^3^Department of Neurosurgery, West Theater General Hospital, Chengdu, Sichuan Province 610000, China

## Abstract

Intracerebral hemorrhage (ICH) is lethal but lacks effective therapies. Nicotinamide adenine dinucleotide (NAD^+^) is a central metabolite indispensable for a broader range of fundamental intracellular biological functions. Reduction of NAD^+^ usually occurs after acute brain insults, and supplementation of NAD^+^ has been proven neuroprotective. P7C3-A20 is a novel compound featuring its ability to facilitate the flux of NAD^+^. In this study, we sought to determine the potential therapeutic value of P7C3-A20 in ICH. In collagenase-induced ICH mouse models, we found that P7C3-A20 treatment could diminish lesion volume, reduce blood-brain barrier (BBB) damage, mitigate brain edema, attenuate neural apoptosis, and improve neurological outcomes after ICH. Further, RNA sequencing and subsequent experiments revealed that ICH-induced neuroinflammation and microglial proinflammatory activities were significantly suppressed following P7C3-A20 treatment. Mitochondrial damage is an important trigger of inflammatory response. We examined mitochondrial morphology and function and found that P7C3-A20 could attenuate OxyHb-induced impairment of mitochondrial dynamics and functions in vitro. Mechanistically, Sirt3, an NAD^+^-dependent deacetylase located in mitochondria, was then found to play a vital role in the protection of P7C3-A20 against mitochondrial damage and inflammatory response. In rescue experiments, P7C3-A20 failed to exert those protective effects in microglia-specific Sirt3 conditional knockout (CKO) mice. Finally, preclinical research revealed a correlation between the plasma NAD^+^ level and the neurological outcome in ICH patients. These results demonstrate that P7C3-A20 is a promising therapeutic agent for neuroinflammatory injury after ICH and exerts protective actions, at least partly, in a Sirt3-dependent manner.

## 1. Introduction

Intracerebral hemorrhage (ICH) leads to severe neurological impairment and high mortality [1–3]. Over 58% of ICH patients die within one year after onset. Moreover, most survivors experience moderate or severe disability in the chronic stage of ICH [4–6]. Both mechanical removal of hematoma and pharmacological intervention for edema formation and intracranial pressure show only limited effectiveness [7–9]. This is largely due to secondary brain injury, which persistently aggravates brain injury, leading to permanent neurological deficits [4]. Therefore, developing new drugs to treat ICH is necessary.

NAD^+^, as a central metabolite, participates in a broad range of fundamental intracellular biological functions, including metabolism, circadian rhythms, aging, immunity, protein deacetylation reactions, and redox homeostasis [10]. Reduced NAD^+^ level is a characteristic manifestation after acute brain insults, such as ischemic stroke, ICH, and traumatic brain injury (TBI), and increasing evidence has proven enhancement of NAD^+^ to be neuroprotective [11–14]. P7C3-A20, a novel aminopropyl carbazole compound, can bind to nicotinamide phosphoribosyl transferase (NAMPT) to enhance the NAD^+^ salvage pathway in normal mammalian cells [15]. Moreover, P7C3-A20 carries favorable pharmacokinetic and pharmacological properties, such as measurable clearance rate, half-life, and bioavailability [11]. Importantly, P7C3-A20 can readily cross the blood-brain barrier with safety even at doses several folds higher than the efficacious dose [16]. Recently, the neuroprotective effects of P7C3-A20 have been demonstrated in several animal models of human diseases [11, 14, 15]. For example, P7C3-A20 mediated the preservation of otherwise depleted neuronal NAD^+^ after traumatic brain injury, which blocked neurodegeneration [16, 17]. In the mice with permanent middle cerebral artery occlusion through electrocoagulation, P7C3-A20 administration could raise NAD^+^ level and reduce infarct size at 24 h after injury [11]. Given the critical role of NAD^+^ in ICH injury and the capability of P7C3-A20 to enhance NAD^+^ production, P7C3-A20 may be suitable for treating ICH.

Sirt3, mainly located in the mitochondria, is an evolutionary conserved NAD^+^-dependent class-III histone deacetylase [18]. Cumulative evidence supports that Sirt3, as a central regulatory hub for mitochondria, has diverse impacts on mitochondrial quality control [19]. Recent studies have indicated that dysregulation of Sirt3 may link mitochondrial dysfunction with the initiation of the inflammatory response during stroke, and that enhancement of Sirt3 deacetylation activities may prevent these pathological injuries [19–21]. Notably, NAD^+^ is required for Sirt3 to maintain deacetylase activity and regulate essential substrates for mitochondrial metabolism [18, 22, 23]. Along with the well-documented role played by Sirt3-related mitochondrial quality control in neuroprotection, it is very worthwhile to explore whether P7C3-A20 protects the post-ICH secondary injury by activating Sirt3-related mitochondrial quality control.

Here, we assessed the protective effects of P7C3-A20 on secondary brain injury in both in vivo and in vitro models of ICH. Moreover, if so, the molecular mechanisms underlying the therapeutic action of P7C3-A20 in ICH were further explored. Additionally, the potential correlation between the plasma NAD^+^ level and the neurological outcome was evaluated in ICH patients.

## 2. Material and Methods

### 2.1. Animals and Ethics

Healthy adult male C57BL/6J mice aged 8 weeks (wild type, 20-25 g) were offered by the Animal Center of the Air Force Medical University, SIRT3-floxp mice by Jackson Laboratory, and CX3cCR1-CreERT2 mice by Gempharmatech Co., Ltd. All mice were housed in an SPF room at 23°C and 60% humidity with a 12 h light/dark cycle, with a 2-week adaptive feeding and free access to food and water before subsequent experiments. All animals received humane care, and all study protocols were approved by institutional or national organs.

### 2.2. ICH Surgery and Mediation

A mouse model was built up through inducing ICH with collagenase, as described previously [24]. Having been anesthetized using 2% pentobarbital sodium, the mouse was fixed with a stereotaxic apparatus. A midline scalp incision from the nasion to the superior nuchal line was made to expose the target position on the calvarium. The skull was drilled to make a 0.5 mm diameter hole allowing for the insertion of a needle into the corpus striatum. The needle was positioned 0.20 mm anterior, 2.30 mm right lateral, and 3.50 mm deep, followed by infusion of saline (0.25 *μ*L volume, containing 0.075 U of collagenase VII-S [C0773, Sigma-Aldrich Co., St. Louis, MO, USA]). Afterward, the needle stayed for 5 min to prevent the possible leakage of the saline. Then, the hole was sealed, and the scalp was closed with sutures. The same procedures, except for the infusion with collagenase, were performed in the sham group. The dead mice (15–20% mortality) or ICH model mice without neurological deficits were excluded from the following analysis. Then, experimental mice were randomly averaged into three groups: sham group, ICH group, and ICH+P7C3-A20 group. At different time points after ICH, the mice in equal numbers were randomly selected from each group and divided into the following experiments for analysis: neurobehavioral tests, quantification of lesion volume, BBB permeability, Western blot analysis, transmission electron microscopy, and immunofluorescence staining.

**Figure 1:**
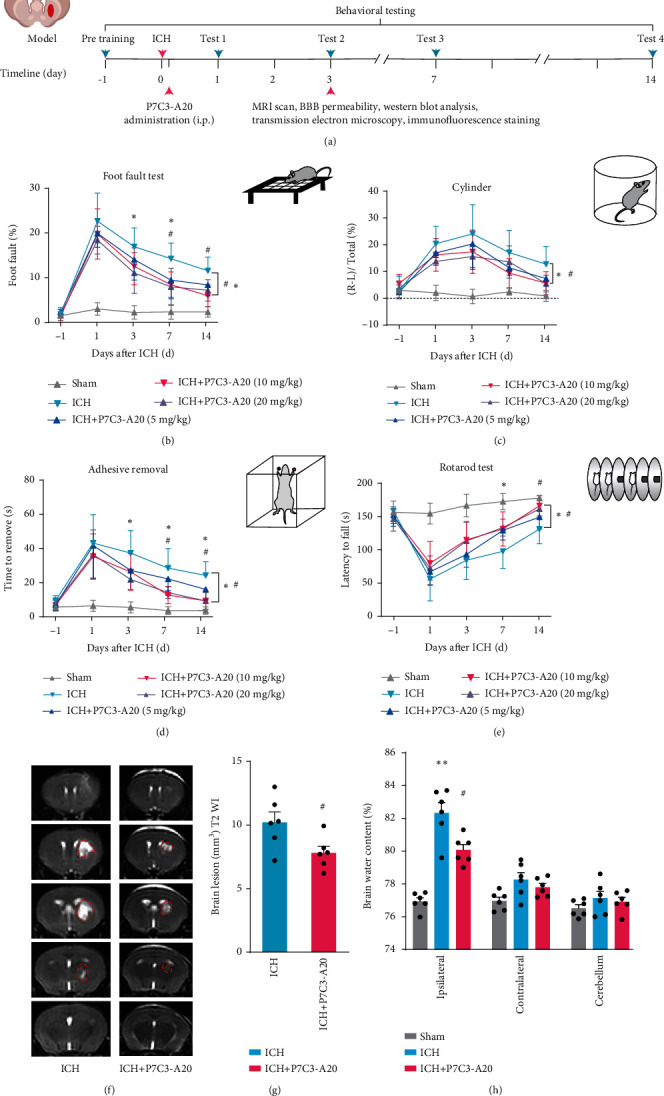
The effects of P7C3-A20 on hemorrhage volume and neurological function after ICH. (a) Experimental schematic. Sensorimotor functions were measured by foot fault (b), cylinder (c), adhesive removal (d), and rotarod test (e) at 1, 3, 7, and 14 d after ICH. ^#^*P* < 0.05, ICH vs. ICH+P7C3-A20 (10 mg/kg); ^∗^*P* < 0.05, ICH vs. ICH+P7C3-A20 (20 mg/kg); *n* = 6. The asterisk (^∗^) and hashtag (^#^) at the right indicate the group differences. The asterisk (^∗^) and hashtag (^#^) on the top indicate single day comparison between the two groups. (f) Representative axial T2 images were obtained from each group at 3 d after ICH. Red lines delineate lesion areas. (g) Quantification of hematoma volumes on T2-weighted images. ^#^*P* < 0.05, ICH vs. ICH+P7C3-A20; *n* = 6. (h) Quantification of brain water content in each group at 3 d after ICH. Ipsilateral: ^∗∗^*P* < 0.01, ICH vs. sham; ^#^*P* < 0.05, ICH vs. ICH+P7C3-A20; *n* = 6. Values are expressed as mean ± SD. Significance was determined by Student's *t*-test (g) and two-way repeated ANOVA with Bonferroni post hoc tests (b–e and h).

As previously described, P7C3-A20 (HY-15978, MCE, Shanghai, China) and vehicle were prepared in DMSO, 5% dextrose, and Kolliphor. According to the results of our preexperiment, 10 mg/kg P7C3-A20 was used as the lowest therapeutic dose. Briefly, the mouse was administered with either 10 mg/kg of P7C3-A20 or vehicle intraperitoneally at 30 min after ICH, using a randomized selection process. All the experimental mice were sacrificed 72 h later for histological and molecular biologic assays or treated with vehicle or P7C3-A20 once daily for behavioral testing (Figure 1(a)).

### 2.3. Quantification of Lesion Volume

A 3T small animal MRI scanner (Bruker MRI GmbH, Germany) was used to produce brain images. T2-weighted images were used to visualize and quantify the ICH brain lesion with the following parameters: repetition time (TR) = 1,000 ms, echo time (TE) = 110.5 ms, image matrix = 336 × 446, field of view (FOV) = 90 × 90 mm^2^, and 0.7 mm slice thickness. Having been anesthetized by the method described above, the mouse was placed in a suitable device over an MRI-compatible holder. Brain tissues were scanned and sliced into equally spaced coronal sections, and each is 0.7 mm thick. The lesion area was calculated using ImageJ by a physician blinded to the experimental grouping. The volumes were then calculated by multiplying the sum of the area by the distance between every two sections.

### 2.4. Brain Water Content Measurement

Brain water content was measured in a wet/dry method previously described [25]. The brain tissues were taken out and quickly divided into three parts: hemorrhage hemispheres, contralateral hemispheres, and cerebellums. Each part was immediately measured for its wet weight, then dried in an oven at 95 to 100°C for 72 h. The part was weighed once more to obtain its dry weight. Tissue water content = ([wet weight − dry weight]/wet weight) × 100%.

### 2.5. Neurobehavioral Tests

#### 2.5.1. Foot Fault Test

The foot fault test was used to assess sensorimotor coordination during spontaneous locomotion, as described previously [9]. The mouse was placed on an elevated metal wire (2.5 mm diameter) grid surface, with a grid opening of 2.25 cm^2^ and videotaped for 1 min from beneath the grid. The videotape was analyzed by a blinded investigator for the number of total steps and foot faults. For each mouse, one foot fault was referred to once misplacing the impaired forepaw or hindpaw into the grid. The percentage of foot faults in total steps made by that paw was calculated. The test was initiated at 1 d before the surgery and repeated at 1, 3, 7, and 14 d after surgery.

#### 2.5.2. Adhesive Removal Test

The adhesive removal test was performed as described previously to evaluate the tactile responses and sensorimotor asymmetries [26]. Tapes (2 mm × 3 mm) were adhered on the left forepaw. The tactile response was described as the time spent removing the adhesive tape from the impaired forepaw with its mouth during a maximum of 120 sec. The test was initiated at 1 d before surgery and repeated at 1, 3, 7, and 14 d after surgery.

#### 2.5.3. Cylinder Test

The cylinder test was conducted as described previously to assess the asymmetry in forepaw use [27]. The mouse was placed in a transparent cylinder and videotaped from the top. Videotapes were analyzed in slow motion for 8 minutes to record forepaw (left/right/both) use during the first contact against the cylinder wall after rearing and during lateral exploration. Uninjured mice typically showed no preference in using two forepaws, whereas injured mice displayed decreased use of the left forepaw, depending on the severity of the injury. Forepaw use asymmetry which was calculated with the following equation: contralateral paw use = (right − left)/(left + right + both) × 100%. The test was initiated at 1 d before surgery and repeated at 1, 3, 7, and 14 d after surgery.

#### 2.5.4. Rotarod Test

The rotarod test was performed as described previously to assess motor coordination [9]. The mouse was placed on an accelerated rotating rod with speed increasing linearly from 4 to 40 rpm within 5 min. At a given day, each mouse was tested 3 times with an intermission of 5 min between tests. The trial ended when the speed of the apparatus reached 40 rpm (5 min) or when the mouse fell off the rod or completed one full revolution on the rod. Latency to fall off the rotating rod was recorded in seconds, with a maximum of 300 s. Data were expressed as mean values from the 3 trials. The test was initiated at 1 d before surgery and repeated at 1, 3, 7, and 14 d after surgery.

### 2.6. BBB Permeability

Evans blue dye extravasation was used to evaluate BBB permeability at 3 d after ICH, as described previously [28]. At 3 h before being sacrificed, the mouse was injected with 2% Evans blue in saline through the tail vein (4 mL/kg), followed by transcardiac perfusion with 40 mL of 0.9% cold saline. Then, the brain tissue was removed, weighed, sliced into 6 equally spaced coronal sections for photographs, homogenized with saline, and centrifugated (12,000 × *g*, 30 min). Next, the trichloroacetic acid in an equal volume was transferred to the resultant supernatant. After overnight incubation (4°C) and another centrifugation (12,000 × *g* at 4°C for 30 min), the supernatant was subjected to spectrophotometrical quantification (620 nM) to measure the extravasation of Evans blue dye.

### 2.7. Tissue Preparation

At post-ICH 72 h, the mouse was given a high dose of 2% pentobarbital sodium. After being sacrificed, the mouse received transcardiac perfusion of 4% paraformaldehyde. The brain tissue was collected, stored in 4% paraformaldehyde overnight at 4°C, then dehydrated with 10%, 20%, and 30% sucrose solutions. The brain tissue was cut into 15-25 *μ*m sections for further experiments and analyses. We selected brain sections at -0.3 to +0.5 mm anterior to bregma. Then, we identified tissue within 1 mm of the hematoma as perihematomal tissue used for immunofluorescence analysis and TUNEL staining [24].

### 2.8. Immunofluorescence Staining

Immunofluorescence staining was performed as described previously [29]. Briefly, the brain tissue was sampled as described above. The 15-25 *μ*m brain sections were obtained, incubated with phosphate buffered saline (PBS) solution comprising 0.1% Triton X-100 for 30 min, and blocked with PBS solution comprising 5% goat serum (16210064, Gibco, NY, USA) for 30 min. Then, the sections were incubated overnight at 4°C with primary antibodies: IBA1 (ab5076, 1 : 500, Abcam, Cambrige, USA) and CD68 (ab6640, 1 : 200, Abcam, Cambrige, USA). After incubation, the sections were rinsed in PBS for 3 × 5 min washes and incubated for 1 h at 25°C with corresponding secondary antibodies: Alexa Fluor 488 antirabbit IgG (A11034, Invitrogen, Carlsbad, CA, USA) and Alexa Fluor 594 antigoat IgG (A11058, Invitrogen, Carlsbad, CA, USA). Lastly, the sections were dyed in DAPI solution (S36939; Invitrogen, Carlsbad, CA, USA) for 15 min at 25°C. The images of all sections were captured blindly using an A1 Si confocal microscope (Nikon, Japan).

### 2.9. Terminal Deoxynucleotidyl Transferase-Mediated dUTP Nick End Labeling (TUNEL) Staining

Cell death in the perihematomal brain tissues was evaluated by a blinded observer using the In Situ Cell Death Detection Kit (Roche, 12156792910, Mannheim, Germany) according to the manufacturer's protocol [29]. After treatment with 0.3% hydrogen peroxide (30 min) and incubation with 0.25% proteinase K (45 min, 37°C), the slices were dyed using TUNEL reaction solution in the dark (1 h, 37°C). Last, the sections were immersed in DAPI for 10 min (37°C). The degree of apoptosis was assessed according to the ratio of TUNEL-positive cells to DAPI-stained cells.

### 2.10. Quantitative Real-Time PCR

Total RNA was extracted with TRIzol Reagent (Invitrogen, 15596026, Carlsbad, California, USA) according to the manufacturer's protocol [24]. Total RNA (1 mg) was reverse-transcribed with HiScript II Q RT SuperMix for quantitative real-time PCR (qRT-PCR) (R223-01, Vazyme, Nanjing, China). An iQTM 5 Optical Module Real-Time PCR Detection System (Bio-Rad, USA) was used to conduct qRT-PCR. ChamQTM SYBR qPCR Master Mix (Q311-02, Vazyme Biotech, Nanjing, China) was used to quantify mRNA according to the manufacturer's instructions. The primers used are listed in Supplementary Table 1. The mRNA levels were normalized to *β*-actin and calculated using 2 − ΔΔCt method.

### 2.11. Cell Culture and Treatment

Mouse BV2 microglia cells (CL-0493; Pricella, Wuhan, China) were purchased from Wuhan Pricella Biotechnology Co., Ltd. BV2 cells were cultured in Dulbecco's modified Eagle's high-glucose medium (11965092, Gibco; Thermo Fisher Scientific, Inc. Waltham, MA, USA) supplemented with 10% fetal bovine serum (15240062, Gibco; Thermo Fisher Scientific, Inc. Waltham, MA, USA), 100 U/mL penicillin, and 100 *μ*g/mL streptomycin (15140122, Gibco; Thermo Fisher Scientific, Inc. Waltham, MA, USA) at 37°C in a 95% humidified 5% CO_2_ cell culture incubator [30]. The control group was exposed to an equivalent volume of PBS and the OxyHb group to 30 *μ*M oxyhemoglobin (OxyHb; O7109, Solarbio, Beijing, China) for 24 h to mimic ICH [31]. The OxyHb+P7C3-A20 group was treated with 60 *μ*M P7C3-A20 for 2 h before OxyHb incubation [32]. The Sirt3 knockout BV2 single-clone cell line was generated using a CRISPR/Cas9 genome-editing approach [33]. Briefly, the cells were transduced with lentivirus for 3 d, then treated with 1 *μ*g/mL puromycin (cat. no. 58-58-2, Sigma-Aldrich, St. Louis, MO, USA) for 1 week. After cloning Sirt3 sgRNAs into lentiCRISPR, positive cells were selected with 200 *μ*g/mL G418 and 1 *μ*g/mL puromycin for 1 week.

### 2.12. Mitochondrial Morphology Staining, Mitochondrial ROS Detection, and Mitochondrial Membrane Potential (MMP) Assay

MitoTracker Red CMXRos solution (Cat.M7512, Life Technologies, Carlsbad, CA, USA) (10 nM) was used to assess mitochondrial morphology [29]. BV2 cells were incubated with 10 nM MitoTracker staining working solution (30 min, 35°C) and washed using PBS thrice. Then, the mitochondria were pictured by a laser-scanning confocal microscope (Nikon, A1Si, Japan), then subjected to ImageJ software to quantify mitochondrial length and width. All the data were shown as aspect ratio.

The level of mitochondrial ROS was measured through staining with 5 nM MitoSOX™ Deep Red Reagent (M36008; Invitrogen, Shanghai, China) for 30 min at 35°C [24]. After washing with Hank's balanced salt solution thrice, the stained samples were imaged through a fluorescence microscope and subjected to the ImageJ software to calculate the relative fluorescence level.

Having been incubated in 10 nM JC-1 (C2006; Beyotime, Shanghai, China) for 30 min at 33°C, the BV2 cells were washed thrice with PBS to remove JC-1 [34]. Then, the cells were observed with the same microscope. The MMP quantification was measured according to their relative fluorescence levels.

### 2.13. Transmission Electron Microscopy

At 3 days after ICH, the mice were anesthetized and perfused as before [35]. The hematoma boundary was first identified by the presence of blood. All perihematoma sample boxes (1 × 1 × 1 mm) were dissected immediately adjacent to the hematoma, with one side of each box aligned with the hematoma boundary. After fixation overnight in 4% glutaraldehyde, the perihematoma brain tissues were postfixed in 1% osmium tetroxide for 1 h, dehydrated using a graded ethanol immersion series, and embedded in resin. The samples were cut into 80 nM sections using an ultramicrotome (Leica, Vienna, Austria). Then, the ultrathin sections were fixed on 200-slot grids coated with pioloform membranes and observed using a JEM-1400 electron microscope (JEOL, Tokyo, Japan).

### 2.14. Western Blot Analysis

We conducted Western blot analysis according to the procedures described previously [24]. An ultrasonic homogenizer was used to homogenize the samples in the lysis buffer comprising 1% protease inhibitor. After lysate centrifugation, the supernatant was collected into a new tube. With its concentration measured using the BCA Protein Assay Kit (UA276918; Thermo Scientific, Waltham, MA, USA), the protein was isolated using sodium dodecyl sulfate-polyacrylamide gel electrophoresis (SDSPAGE) (30 *μ*g of protein per well of the electrophoresis gel), then transferred to polyvinylidene fluoride (PVDF) membranes (IPVH-00010, Millipore Corporation, Billerica, MA, USA). Having been blocked by 5% nonfat milk in Trisbuffered Saline and Tween 20 (TBST) (pH 7.6), the membranes were incubated with the following primary antibodies: anticlaudin-5 (1 : 800, ab131259, Abcam, Cambridge, MA, USA); antioccludin (1 : 1000, ab216327, Abcam, Cambridge, MA, USA); anti-Bax (1 : 1000, 50599, Proteintech, Wuhan, Chin); anti-Bcl2 (1 : 1000, 12789, Proteintech, Wuhan, Chin); anti-ZO-1 (1 : 500, ab96587, Abcam, Cambridge, MA, USA); anti-*β*-actin (1 : 3000, AC026, ABclonal, Wuhan, China). Then, the membranes were incubated with the horseradish peroxidase-conjugated secondary antibodies till to be washed with TBST three times, 5 min each time. Finally, the protein bands were detected using a Bio-Rad imaging system (Bio-Rad, Hercules, CA, USA).

### 2.15. Patients

This study was approved by the Ethics Committee of Tangdu Hospital, Fourth Military Medical University (Protocol ID number: TDLL-202210-08) and was carried out from June 2021 to June 2022. A total of 76 mild or moderate ICH patients aged 18-85 years managed nonoperatively were enrolled in the study. Excluded were those who had received ICH surgery or died during the follow -up, as well as those with disabling neurological diseases or serious systemic diseases (such as uremia, cirrhosis, or malignancy). The modified Rankin Scale (mRS) score was used to assess functional outcomes at 3 months and divided into favorable (mRS score = 0 − 2) or poor (mRS score = 3 − 5) as described previously [36].

### 2.16. Statistical Analysis

GraphPad Prism 8.0 software (La Jolla, CA, USA) and SPSS 20.0 (SPSS Science Inc., Chicago, IL, USA) were used for statistical analysis [37]. Normal distribution was assessed with the Shapiro-Wilk normality test or normal Q-Q plots. All the data were expressed as mean ± standard deviation (SD) unless otherwise noted. Unpaired two-tailed Student's *t*-test (for two independent groups) or ordinary one-way ANOVA with Bonferroni post hoc test (for more than two groups) was used to test statistical significance between groups for data with normal distribution and equal variance; alternatively, *t*-test or one-way ANOVA with Welch's correction was used for normally distributed data with unequal variances. Besides, two-way repeated ANOVA with Bonferroni post hoc test was used for behavioral analysis [9]. Independent risk factors for poor outcomes were confirmed by multivariate logistic regression using SPSS 20.0 [34]. *p* < 0.05 was considered statistically significant. More details on the statistical tests and the methods used in our study were listed in Supplementary Table 2.

## 3. Results

### 3.1. Effects of P7C3-A20 on Lesion Volume and Neurological Function after ICH

Based on previous studies regarding the treatment of P7C3-A20 on CNS diseases, we evaluated the protective effects of three different doses (5 mg/kg, 10 mg/kg, and 20 mg/kg) of P7C3-A20 on sensorimotor ability after ICH [16, 38, 39]. Behavioral tests were conducted at 1, 3, 7, and 14 d after surgery, including foot faults (Figure 1(b)), cylinder (Figure 1(c)), adhesive removal (Figure 1(d)), and rotarod tests (Figure 1(e)). Results showed that 10 mg/kg and 20 mg/kg P7C3-A20 exhibited more noticeable improvement in the sensorimotor ability of mice after ICH than 5 mg/kg P7C3-A20, with statistical significance. Notably, there was no significant difference between the effects of 10 and 20 mg/kg P7C3-A20 after ICH. Specifically, As shown in Figure 1(b), foot faults were significantly elevated in the ICH group, compared to that in sham group (*F* [4, 25] = 22.84, *p* < 0.0001), while 10 and 20 mg/kg P7C3-A20 treatment significantly reduced foot faults at 1, 3, 7, and 14 d after ICH surgery in the grid-walking test (ICH vs. ICH+P7C3-A20 [10 mg/kg], *F* [4, 25] = 22.84, *p* = 0.0436; ICH vs. ICH+P7C3-A20 [20 mg/kg], *F* [4, 25] = 22.84, *p* = 0.0225). Comparable outcomes were found in the cylinder test in which ICH group shows obviously increased bias, compared to that in sham group (*F* [4, 25] = 23.81, *p* < 0.0001), while 10 and 20 mg/kg P7C3-A20 treatment markedly reduced bias at 1, 3, 7, and 14 d after ICH (ICH vs. ICH+P7C3-A20 [10 mg/kg], *F* [4, 25] = 23.81, *p* = 0.0418; ICH vs. ICH+P7C3-A20 [20 mg/kg], *F* [4, 25] = 23.81, *p* = 0.0212) (Figure 1(c)). This therapeutic effect was also proven in the adhesive removal test and latency to fall test (adhesive removal: sham vs. ICH, *F* [4, 25] = 18.07, *p* < 0.0001; ICH vs. ICH+P7C3-A20 [10 mg/kg], *F* [4, 25] = 18.07, *p* = 0.0135; ICH vs. ICH+P7C3-A20 [20 mg/kg], *F* [4, 25] = 18.07, *p* = 0.0115; latency to fall: sham vs. ICH, *F* [4, 25] = 26.11, *p* < 0.0001; ICH vs. ICH+P7C3-A20 [10 mg/kg], *F* [4, 25] = 26.11, *p* = 0.0108; ICH vs. ICH+P7C3-A20 [20 mg/kg], *F* [4, 25] = 26.11, *p* = 0.0368) (Figures 1(e) and 1(e)). So, according to above results, 10 mg/kg P7C3-A20 was used as the lowest therapeutic dose for subsequent experiments. To verify the effect of P7C3-A20 on lesion volume, MRI was performed at 3 d. MRI results indicated that compared to the ICH group, there was a significant decrease in lesion volume in ICH mice treated with P7C3-A20 (t [10] = 2.473, *p* = 0.0329) (Figures 1(f), and 1(g)). Collectively, these results showed that P7C3-A20 reduced lesion volume and promoted the recovery of neurological function after ICH.

### 3.2. P7C3-A20 Ameliorated BBB Impairment, Brain Edema, and Neural Apoptosis after ICH

**Figure 2:**
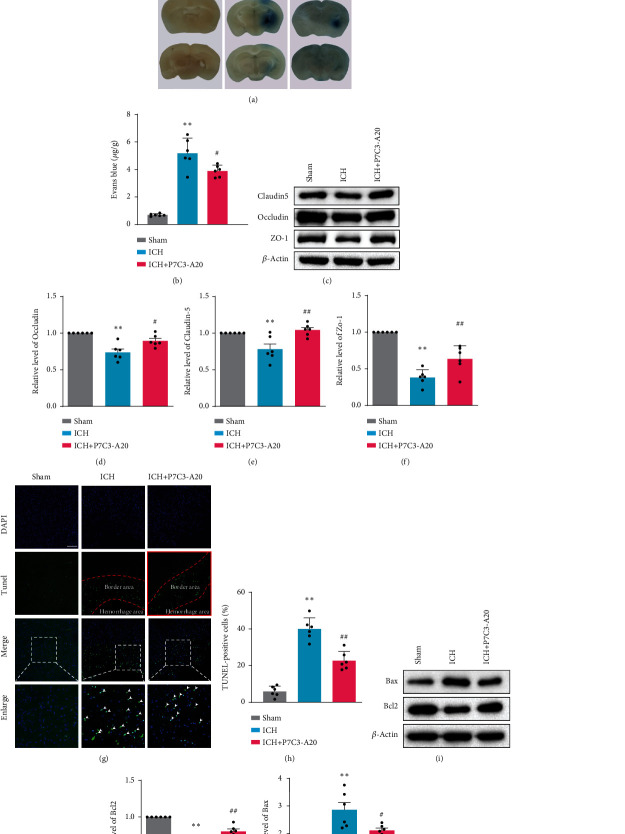
P7C3-A20 ameliorated BBB impairment, brain edema, and neural apoptosis after ICH. (a) Representative brain sections with Evans blue showing different dye extravasation in each group at 3 d after ICH. (b) Quantification of Evans blue in each group. ^∗∗^*P* < 0.01, ICH vs. sham; ^#^*P* < 0.05, ICH vs. ICH+P7C3-A20; *n* = 6. (c–f) Western blot assay to detect the expression of claudin-5, occludin, and ZO-1 in each group and the related statistical analysis. ^∗∗^*P* < 0.01, ICH vs. sham; ^#^*P* < 0.05 and ^##^*P* < 0.01, ICH vs. ICH+P7C3-A20; *n* = 6. (g) Representative TUNEL staining images. The area between the two red lines indicates the peripheral region of the hematoma. Each micrograph next to the boxed area shows an amplified version of the white square, in which white arrowheads indicate TUNEL-positive cells. (h) Quantitative analyses of TUNEL-positive cells in the perihematoma brain tissues in each group at 3 d after ICH. Scale bar, 100 *μ*m. ^∗∗^*P* < 0.01, ICH vs. sham; ^##^*P* < 0.01, ICH vs. ICH+P7C3-A20; *n* = 6. (i–k) Representative Western blot images of Bax and Bcl-2. The densities of the protein bands were analyzed and normalized to *β*-actin. ^∗∗^*P* < 0.01 ICH vs. sham; ^#^*P* < 0.05 and ^##^*P* < 0.01 ICH vs. ICH+P7C3-A20; *n* = 6. Values are mean ± SD. Significance was determined by one-way ANOVA with Bonferroni post hoc tests (b, d, e, f, h, j, and k).

BBB impairment is a critical process in ICH-induced secondary brain injury. ICH induced an obvious elevation in Evans blue extravasation in brain tissue samples compared to the sham group (*F* [2, 15] = 69.01, *p* < 0.0001), but the ICH+P7C3-A20 group significantly inhibited this elevation compared to the ICH group (*F* [2, 15] = 69.01, *p* = 0.0155) (Figures 2(a) and 2(b)), indicating that P7C3-A20 could protect BBB integrity against ICH injury. In Western blot results for BBB-related proteins, we further found that compared to sham, the ICH group decreased the expression of tight junction proteins (occludin, claudin-5, and ZO-1) (occludin, *F* [2, 15] = 16.55, *p* < 0.0001; claudin-5, *F* [2, 15] = 14.70, *p* = 0.0004; Zo-1, *F* [2, 15] = 39.38, *p* = 0.0004), which was also reversed by additional P7C3-A20 treatment (occludin, *F* [2, 15] = 16.55, *p* = 0.0118; claudin-5, *F* [2, 15] = 14.70, *p* = 0.0025; Zo-1, *F* [2, 15] = 39.38, *p* = 0.0067) (Figures 2(c)–2(f)). In line with this, we found P7C3-A20 also significantly ameliorated brain edema after ICH (ipsilateral: sham vs. ICH, *F* [2, 15] = 28.74, *p* < 0.0001, ICH vs. ICH+P7C3-A20, *F* [2, 15] = 28.74, *p* = 0.0415) (Figure 1(h)).

Then, the effect of P7C3-A20 on neural apoptosis was measured by TUNEL staining after ICH. Compared to the sham, the ICH group significantly increased the levels of positive cells (*F* [2, 15] = 73.49, *p* < 0.0001), while the ICH+P7C3-A20 group repressed cell apoptosis compared to the ICH group (*F* [2, 15] = 73.49, *p* < 0.0001) (Figures 2(f) and 2(i)). Meanwhile, Western blotting displayed an evident rise of Bax and fall of Bcl2 in the ICH group compared to sham group (Bax, *F* [2, 15] = 33.58, *p* < 0.0001; Bcl2, *F* [2, 15] = 90.40, *p* < 0.0001), whereas both were significantly reversed by additional P7C3-A20 treatment (Bax, *F* [2, 15] = 33.58, *p* = 0.0153; Bcl2, *F* [2, 15] = 90.40, *p* < 0.0001) (Figures 2(g), 2(h), and 2(j)).

### 3.3. P7C3-A20 Suppressed Microglial Activation and Attenuated Neuroinflammation after ICH

**Figure 3:**
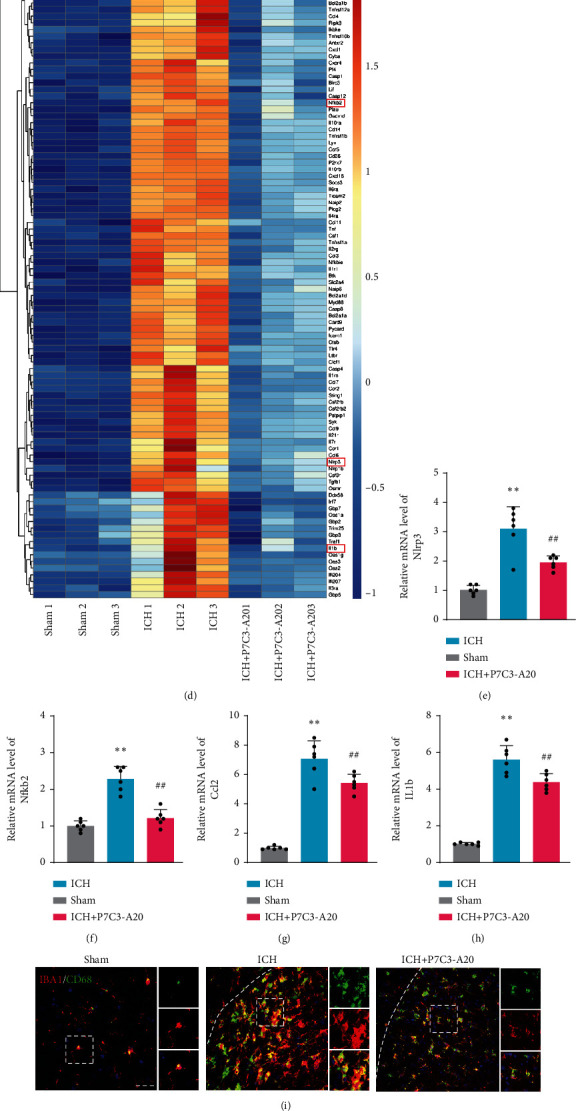
P7C3-A20 attenuated neuroinflammation injury and suppressed microglial proinflammatory activities after ICH. (a) Different gene distribution was clearly seen on the volcano plot. (b) Venn diagram showed that the expression of 1047 overlapped genes was reversed by P7C3-A20 treatment. (c) KEGG enrichment analysis of the overlap of genes in the Venn diagram. (d) The heat map showed the changing trend in each group's expression of inflammation-related genes. (e–h) Representative inflammatory cytokines (Nfkb2, Nlrp3, IL-1b, and Ccl2) from upstream and downstream inflammation pathways were assayed by qPCR to verify the accuracy of RNA sequencing results. ^∗∗^*P* < 0.01, ICH vs. sham; ^##^*P* < 0.01, ICH vs. ICH+P7C3-A20; *n* = 6. (i) Immunofluorescence staining for Iba1 (red) with CD68 (green) revealing the activated microglia levels in each group. The white lines represent the edges of the hemorrhage. Each micrograph next to the boxed area shows an amplified version of the white square with different fluorescent labels. Scale bar, 50 *μ*m. (j) Representative immunofluorescence staining of Iba1-positive cells within the peripheral tissue in each group and skeletonized analysis. Scale bar, 20 *μ*m. (k–n) Statistical analysis of branch numbers, total branch length (micrometers), average branch length (micrometers), and soma area (square micrometers) of Iba1-positive cells in each group. ^∗∗^*P* < 0.01 ICH vs. sham; ^#^*P* < 0.05 and ^##^*P* < 0.01, ICH vs. ICH+P7C3-A20 (*n* = 20 from 6 mice per group). Values are mean ± SD. Significance was determined by one-way ANOVA with Bonferroni post hoc tests (e, f, g, k, l, and m) or with Welch's correction (h, n).

We employed RNA sequencing (RNA-seq) to profile the mRNA expression ensuing P7C3-A20 treatment. Different gene distributions could be clearly seen on the volcano plot (Figure 3(a)). The volcano plot showed that compared to the sham group, the ICH group contained 2303 upregulated and 309 downregulated genes, and that the ICH+P7C3-A20 group contained 222 upregulated and 1060 downregulated genes, compared to the ICH group. Venn diagram showed that the expression of 1047 overlapped genes was reversed by P7C3-A20 treatment (Figure 3(b)). GO analysis revealed critical biological processes enriched in inflammatory response (Supplementary Figure 1). As shown in Figure 3(c), KEGG analysis demonstrated that the expression levels of genes involved in inflammation-related biological processes (cytokine-cytokine receptor interaction, NF-kappa B signaling pathway, and NOD-like receptor signaling pathway) were greatly altered after P7C3-A20 treatment, suggesting that P7C3-A20 might regulate inflammatory response to exert its protective effect. Then, a heat map presented the trends in the expression of genes involved in cytokine-cytokine receptor interaction, NF-kappa B signaling pathway, and NOD-like receptor signaling pathway (Figure 3(d)). Many inflammation pathway-related genes were changed after P7C3-A20 treatment, including upstream regulators of inflammation pathway, such as Nfkb2 and Nlrp3, and downstream inflammatory cytokines, such as IL-1b and Ccl2. The four inflammatory factors, which could reflect the overall state of neuroinflammation to some extent, were then selected for further study. As shown by the qPCR results (Figures 3(e)–3(h)), ICH group markedly increased Nfkb2, Nlrp3, IL-1b, and Ccl2 at the transcriptional level compared to sham group (Nlrp3, *F* [2, 15] = 33.58, *p* < 0.0001; Nfkb2, *F* [2, 15] = 45.35, *p* < 0.0001; Ccl2, *F* [2, 15] = 94.06, *p* < 0.0001; IL1b, *F* [2, 15] = 234.4, *p* < 0.0001), whereas ICH+P7C3-A20 group obviously decreased those inflammatory factors (Nlrp3, *F* [2, 15] = 33.58, *p* = 0.0019; Nfkb2, *F* [2, 15] = 45.35, *p* < 0.0001; Ccl2, *F* [2, 15] = 94.06, *p* = 0.0079; IL1b, *F* [2, 15] = 234.4, *p* = 0.0026), indicating P7C3-A20 significantly suppressed the ICH-induced activation of inflammatory factors. Since microglia, the dominant immune cells in the central nervous system, participate in the activation and regulation of neuroinflammation after ICH, we then investigated microglial proinflammatory activities. First, the neuroprotective effects of the different doses of P7C3-A20 on neuroinflammatory injury after ICH were evaluated by immunofluorescence staining for Iba1 with CD68, which is consistent with the above results of behavioral tests (Supplementary Figure 2). The density of CD68, a marker indicating microglial hyperactivation, increased obviously after ICH, which was reversed after PC3-A20 treatment (Figure 3(i)). Meanwhile, the morphological and fractal analysis of microglia by Iba1 staining showed that after ICH, the microglia displayed strong retraction, hypertrophic morphology, and augmented cell size, suggesting significant polarization towards activation, while ICH+P7C3-A20 group significantly reversed this trend (Figure 3(j)). Furthermore, we delineated and skeletonized microglial morphology by Iba1 staining using the ImageJ software. ICH group markedly decreased the branch numbers, total branch length, and average branch length and increased the soma area of the microglia compared to sham group (branch number, *F* [2, 15] = 6.077, *p* = 0.0058; total branch length, *F* [2, 15] = 10.72, *p* < 0.0001; average branch length, *F* [2, 15] = 14.85, *p* < 0.0001; soma area, *F* [2, 15] = 234.4, *p* < 0.0001) (Figures 3(k)–3(n)), while P7C3-A20 treatment significantly rescued ICH-induced morphological changes (branch number, *F* [2, 15] = 6.077, *p* = 0.0262; total branch length, *F* [2, 15] = 10.72, *p* = 0.0332; average branch length, *F* [2, 15] = 14.85, *p* < 0.0026; soma area, *F* [2, 15] = 234.4, *p* < 0.0001). By the qPCR, we further evaluated the microglia-specific markers (CD11b, INOS, CD206, and Arg1) to determine the microglial activation state (Supplementary Figure 3). All these results indicated that PC3-A20 suppressed microglial proinflammatory activities and attenuated neuroinflammation injury after ICH.

### 3.4. P7C3-A20 Reversed NAD^+^ Depletion and Alleviated Morphological and Functional Damage of Mitochondria in the Microglia in a Sirt3-Dependent Manner

**Figure 4:**
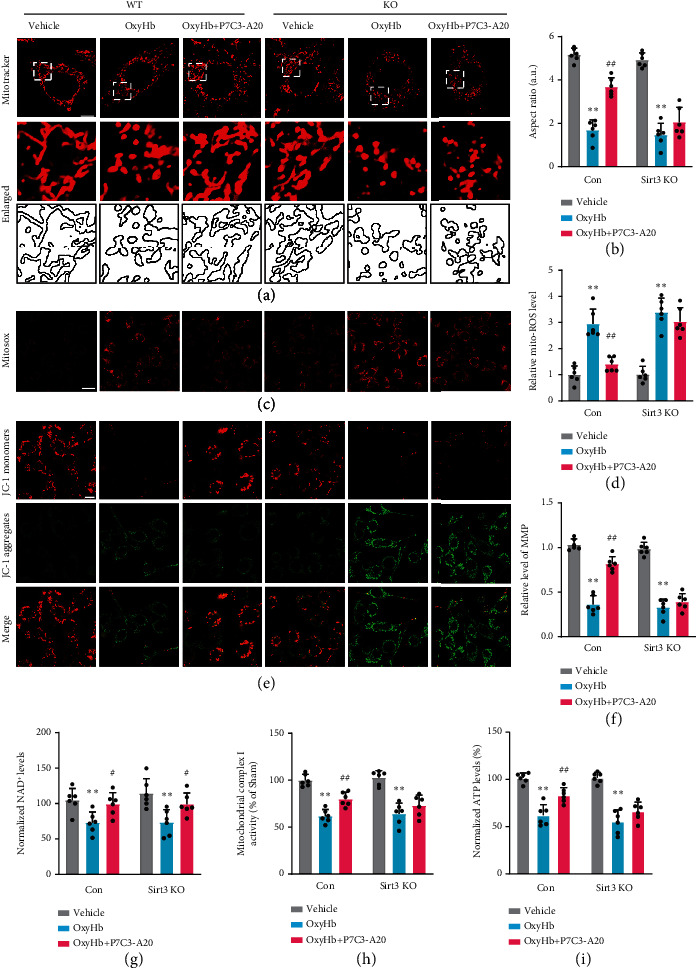
P7C3-A20 reversed NAD^+^ depletion and alleviated morphological and functional damage of mitochondria in the microglia in a Sirt3-dependent manner. (a, b) Changes and quantifications of mitochondrial morphology by MitoTracker staining in BV2 line of control (Con) and Sirt3 knockout after different treatments, respectively. Each micrograph beside the boxed area shows an amplified version of the white square. Scale bar, 10 *μ*m. The Sirt3 knockout neutralized the P7C3-A20-induced increase of the aspect ratio of mitochondria after OxyHb treatment. Con: ^∗∗^*P* < 0.01, OxyHb vs. vehicle; ^##^*P* < 0.01, OxyHb vs. OxyHb+P7C3-A20; *n* = 6; Sirt3 KO: ^∗∗^*P* < 0.01, OxyHb vs. vehicle; *n* = 6. (c, d) Measurement of mitochondrial ROS generation via MitoSOX Deep Red staining. Scale bar, 20 *μ*m. The Sirt3 knockout counteracted the P7C3-A20-mediated suppression of ROS production in mitochondria after OxyHb treatment. Con: ^∗∗^*P* < 0.01, OxyHb vs. vehicle; ^##^*P* < 0.01, OxyHb vs. OxyHb+P7C3-A20; *n* = 6; Sirt3 KO: ^∗∗^*P* < 0.01, OxyHb vs. vehicle; *n* = 6. (e, f) Analysis in MMP by fluorescence staining of JC-1 aggregates (red)/JC-1 monomers (green) illustrating that the Sirt3 knockout neutralized P7C3-A20-induced elevation of MMP after OxyHb treatment. Scale bar, 20 *μ*m. Con: ^∗∗^*P* < 0.01, OxyHb vs. vehicle; ^##^*P* < 0.01, OxyHb vs. OxyHb+P7C3-A20; *n* = 6; Sirt3 KO: ^∗∗^*P* < 0.01, OxyHb vs. vehicle, *n* = 6. (g) Effects of P7C3-A20 on NAD^+^ production in OxyHb-treated BV2 cells of control and Sirt3 knockout, indicating that P7C3-A20 could enhance the flux of NAD^+^ both in Sirt3 knockout and control BV2 cell. (Con: ^∗∗^*P* < 0.01, OxyHb vs. vehicle; ^#^*P* < 0.05, OxyHb vs. OxyHb+P7C3-A20; *n* = 6; Sirt3 KO: ^∗∗^*P* < 0.01, OxyHb vs. vehicle, ^#^*P* < 0.05, OxyHb vs. OxyHb+P7C3-A20; *n* = 6). Other mitochondrial bioenergetics, including mitochondrial complex I activity (h) and ATP levels (i), were quantified (Con: ^∗∗^*P* < 0.01, OxyHb vs. vehicle; ^##^*P* < 0.01, OxyHb vs. OxyHb+P7C3-A20; *n* = 6; Sirt3 KO: ^∗∗^*P* < 0.01, OxyHb vs. vehicle; *n* = 6), all indicating that Sirt3 knockout countered the protective effects of P7C3-A20 on mitochondrial quality control after OxyHb treatment. Values are expressed as mean ± SD. Significance was determined by two-way repeated ANOVA with Bonferroni post hoc tests (b, d, f, g, h, and i).

Mitochondrial damage is a vital inflammation trigger. In our preexperiment, we evaluated mitophagy by analyzing the markers (Tom20, Parkin, and PINK1) of mitophagy using Western blot experiments (Supplementary Figure 4). The results suggested that P7C3-A20 did not affect mitochondrial mitophagy. Therefore, we then turned to investigate the experiment about mitochondrial morphology and function. We explored how P7C3-A20 changes mitochondrial morphology in the BV2 cell line after ICH by MitoTracker staining. The BV2 was exposed to 30 *μ*M OxyHb for 24 h to mimic ICH as described above. We found that OxyHb significantly increased mitochondrial fragmentation, compared to that in the vehicle group (*F* [2, 15] = 358.8, *p* < 0.0001), while this increase was significantly inhibited by P7C3-A20 treatment (OxyHb vs. OxyHb+P7C3-A20, *F* [2, 15] = 358.8, *p* < 0.0001), indicating that P7C3-A20 could help to maintain mitochondrial morphology integrity after ICH (Figures 4(a) and 4(b)). Meanwhile, the effects of P7C3-A20 treatment on mitochondrial function were explored. MitoSOX staining exhibited that compared to that in vehicle group, ROS generation increased markedly in the OxyHb group (*F* [2, 15] = 49.14, *p* < 0.0001), while the OxyHb+P7C3-A20 group reduced the overproduction of mitochondrial ROS, compared to that in the OxyHb group (*F* [2, 15] = 49.14, *p* < 0.0001) (Figures 4(c) and 4(d)). The MMP was assessed according to relative fluorescence levels assayed by JC-1 staining (Figures 4(e) and 4(f)). We observed that the MMP was decreased in the OxyHb group, compared to that in the vehicle group (*F* [2, 15] = 143.8, *p* < 0.0001), while OxyHb+PC3-A20 reversed the downregulation of MMP after OxyHb treatment (*F* [2, 15] = 143.8, *p* < 0.0001). Meanwhile, similar results were observed that OxyHb obviously decreased ATP levels and mitochondrial complex I activities (Figures 4(h) and 4(i)), whereas these changes were reversed by P7C3-A20 treatment (ATP level, OxyHb vs. vehicle, *F* [2, 15] = 56.50, *p* < 0.0001, OxyHb vs. OxyHb+P7C3-A20, *F* [2, 15] = 56.50, *p* = 0.0091; mitochondrial complex I activities, OxyHb vs. vehicle, *F* [2, 15] = 78.55, *p* < 0.0001, OxyHb vs. OxyHb+P7C3-A20, *F* [2, 15] = 78.55, *p* = 0.0035). Those results suggested that P7C3-A20 could alleviate mitochondrial dysfunction after ICH.

Mechanistically, P7C3-A20 can bind to NAMPT, a rate-limiting enzyme in NAD^+^ salvage, and enhance the flux of the NAD^+^ salvage pathway in normal mammalian cells [15]. To assess the effects of P7C3-A20 on NAD^+^ production in BV2, we measured relative NAD^+^ levels in each group (Figure 4(g)). The results showed that OxyHb treatment significantly decreased NAD^+^ levels, while P7C3-A20 could reverse this OxyHb-induced NAD^+^ depletion remarkably (OxyHb vs. vehicle, *F* [2, 15] = 12.74, *p* = 0.0098, OxyHb vs. OxyHb+P7C3-A20, *F* [2, 15] = 12.74, *p* = 0.0390). Since Sirt3 is an NAD^+^-dependent deacetylase essential for mitochondrial function and energy metabolism [23], we speculated that P7C3-A20 might exert protective actions on mitochondria in a Sirt3-dependent manner. To investigate the role of Sirt3 in the protective effect of P7C3-A20 on mitochondria, we knocked out the Sirt3 in BV2 cells. First, P7C3-A20 also reversed NAD^+^ depletion after OxyHb treatment in Sirt3 KO BV2 (OxyHb vs. vehicle, *F* [2, 15] = 12.74, *p* = 0.0008; OxyHb vs. OxyHb+P7C3-A20, *F* [2, 15] = 12.74, *p* = 0.0446), indicating P7C3-A20 could also mediate the enhancement of NAD^+^ in Sirt3 knockout models of BV2 cells, and this enhancement was not affected by Sirt3 protein. Notably, we further found that the protective effect of P7C3-A20 on OxyHb-induced mitochondrial morphology damage was obviously counteracted in knockout groups, compared to that in nonknockout groups (Sirt3 KO: OxyHb vs. vehicle, *F* [2, 15] = 358.8, *p* < 0.0001, OxyHb vs. OxyHb+P7C3-A20, *F* [2, 15] = 358.8, *p* = 0.1149) (Figures 4(a) and 4(b)). Meanwhile, the indexes of mitochondrial bioenergetics, such as mitochondrial ROS production, MMP, ATP production, and mitochondrial complex I activities, were also investigated (Figures 4(c)–4(f), 4(h), and 4(i)). The same changes in those indexes of mitochondrial bioenergetics were found. P7C3-A20 did not show evident protective actions in knockout groups, compared to that in nonknockout groups (Sirt3 KO: ROS, OxyHb vs. vehicle, *F* [2, 15] = 49.14, *p* < 0.0001, OxyHb vs. OxyHb+P7C3-A20, *F* [2, 15] = 49.14, *p* = 0.5408; Sirt3 KO: MMP, OxyHb vs. vehicle, *F* [2, 15] = 143.8, *p* < 0.0001, OxyHb vs. OxyHb+P7C3-A20, *F* [2, 15] = 143.8, *p* = 0.6212; Sirt3 KO: ATP, OxyHb vs. vehicle, *F* [2, 15] = 56.50, *p* < 0.0001, OxyHb vs. OxyHb+P7C3-A20, *F* [2, 15] = 56.50, *p* = 0.4407; Sirt3 KO: mitochondrial complex I, OxyHb vs. vehicle, *F* [2, 15] = 78.55, *p* < 0.0001, OxyHb vs. OxyHb+P7C3-A20, *F* [2, 15] = 78.55, *p* = 0.2849). These results indicated that P7C3-A20 alleviated mitochondrial morphological damage and protected mitochondrial function in a Sirt3-dependent manner.

### 3.5. Microglial Sirt3 CKO Weakened the Protective Effect of P7C3-A20 on the Mitochondrial Ultrastructure and Function in Microglia after ICH in Mice

**Figure 5:**
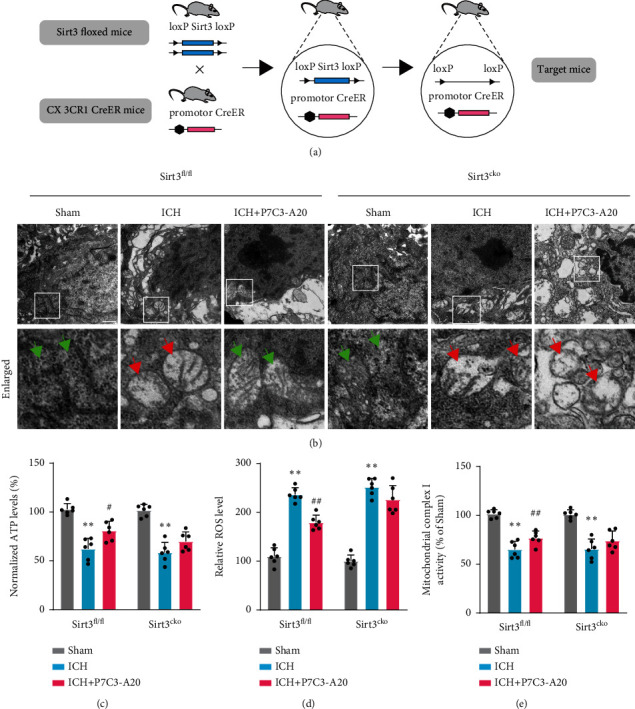
Microglial Sirt3 CKO diminished the protective effect of P7C3-A20 on the mitochondrial ultrastructure and function in microglia after ICH in mice. (a) Flow diagram of the Sirt3 CKO model. Microglia-specific Sirt3 CKO mice were generated by crossbreeding Sirt3^fl/fl^ mice with CX3CR1-CreERT2 mice. (b) Electron microscopy images revealing mitochondrial ultrastructure in microglia. Each micrograph next to the boxed area shows an amplified version of the white square, in which green arrows indicate the normal mitochondria with well-defined cristae, and red arrows indicate the abnormal mitochondria with loss of cristae and swelling. Scale bar, 50 nM. (c-e) Histograms illustrating ATP levels, ROS production, and mitochondrial complex I activities in Sirt3^fl/fl^ and Sirt3^CKO^ mice after different treatments. Sirt3^fl/fl^: ^∗∗^*P* < 0.01, ICH vs. sham; ^##^*P* < 0.01, ICH vs. ICH+P7C3-A20; *n* = 6; Sirt3^CKO^: ^∗∗^*P* < 0.01, ICH vs. sham; *n* = 6. Values are expressed as mean ± SD. Significance was determined by two-way repeated ANOVA with Bonferroni post hoc tests (c, d, and e).

To further confirm the role of Sirt3 in the protective effect of P7C3-A20, microglia-specific Sirt3 CKO mice were generated by crossbreeding Sirt3^fl/fl^ mice with CX3CR1-CreERT2 mice (Figure 5(a)). In these mice, tamoxifen induced Sirt3 CKO in the microglia. Mitochondrial ultrastructure in microglia was observed using transmission electron microscopy after ICH. The sham group exhibited large and mostly oval mitochondria with a folded inner membrane protruding inward to form the crista and an intact outer membrane covering it (Figure 5(b)). And the ICH groups featured mitochondrial swelling, the mitochondrial membrane disruption, and loss of mitochondrial cristae, which is relieved by additional P7C3-A20 treatment (Figure 5(b)). While, compared with control littermates, the protective effect of P7C3-A20 on mitochondrial ultrastructure was partially suppressed in Sirt3 CKO mice (Figure 5(b)). Meanwhile, the indexes of mitochondrial bioenergetics, such as ROS production, ATP production, and mitochondrial complex I activities, were also explored (Figures 5(c)–5(e)). We found that P7C3-A20 exerted protective effects on those indexes of mitochondrial bioenergetics in Sirt3^fl/fl^ mice after ICH, but not in Sirt3 CKO mice (ROS, *F* [2, 15] = 124.5, Sirt3^fl/fl^: ICH vs. ICH+P7C3-A20, *p* < 0.0001, Sirt3^CKO^: ICH vs. ICH+P7C3-A20 *p* = 0.07; ATP, *F* [2, 15] = 44.82, Sirt3^fl/fl^: ICH vs. ICH+P7C3-A20, *p* = 0.0040, Sirt3^CKO^: ICH vs. ICH+P7C3-A20, *p* = 0.1080; mitochondrial complex I, *F* [2, 15] = 104.5, Sirt3^fl/fl^: ICH vs. ICH+P7C3-A20 *p* = 0.0419, Sirt3^CKO^: ICH vs. ICH+P7C3-A20, *p* = 0.1963). All these results suggested that the Sirt3 CKO countered the protective effect of P7C3-A20 on the mitochondrial ultrastructure and function in microglia after ICH in mice.

### 3.6. Microglial Sirt3 Knockout Countered the Protective Effect of P7C3-A20 on the Neuroinflammation after ICH in Mice

**Figure 6:**
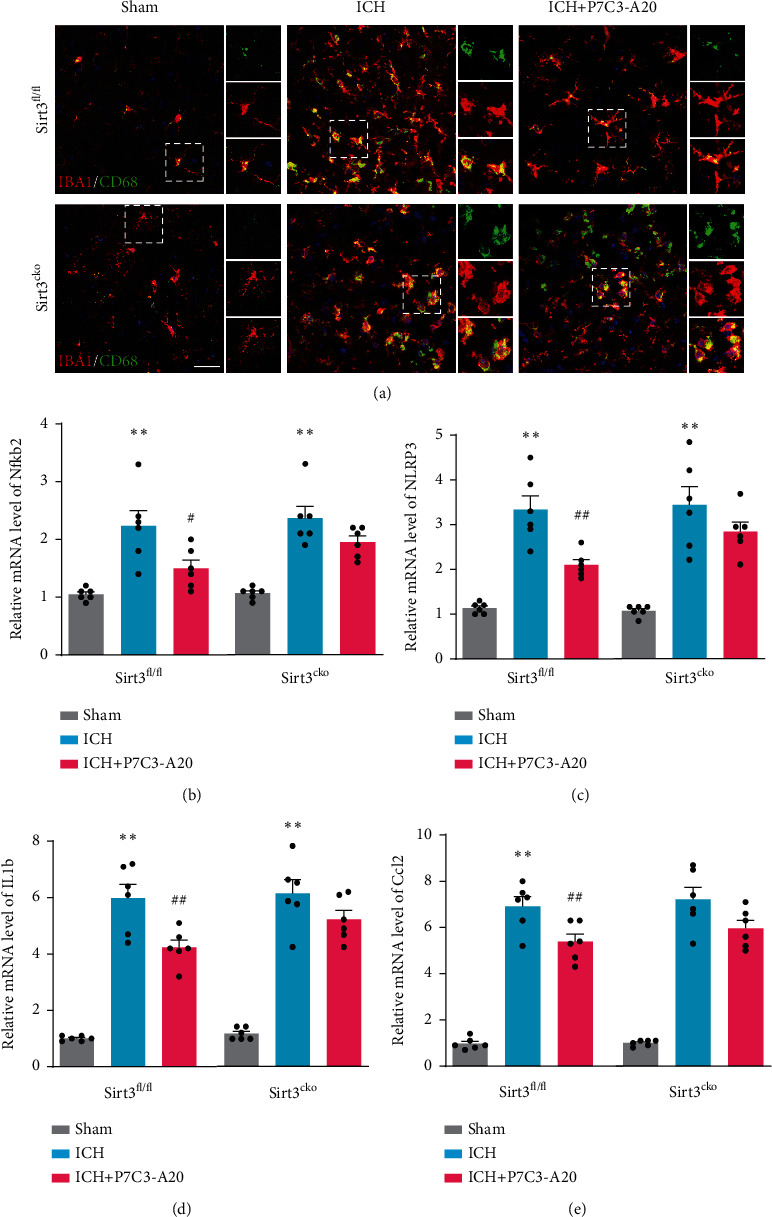
Microglial Sirt3 knockout encountered the protective effects of P7C3-A20 on neuroinflammation in mice. (a) Immunofluorescence staining for Iba1 (red) with CD68 (green) in Sirt3^fl/fl^ and Sirt3 CKO mice after different treatments, respectively, revealing the levels of activated microglia in each group. Each micrograph next to the boxed area shows an amplified version of the white square with different fluorescent labels. Scale bar, 50 *μ*m. (b–e) qPCR quantification of Nfkb2, Nlrp3, IL-1b, and Ccl2 mRNA, revealing the role of Sirt3 in the effect of P7C3-A20 on inflammatory-related mRNA expression. Sirt3^fl/fl^: ^∗∗^*P* < 0.01, ICH vs. sham; ^##^*P* < 0.01, ICH vs. ICH+P7C3-A20; *n* = 6; Sirt3^CKO^: ^∗∗^*P* < 0.01, ICH vs. sham; *n* = 6. Values are expressed as mean ± SD. Significance was determined by two-way repeated ANOVA with Bonferroni post hoc tests (b, c, d, and e).

To confirm the role of Sirt3 in the anti-inflammatory properties of P7C3-A20, we measured microglial proinflammatory activities and expression of the inflammatory-related mRNA in both Sirt3 CKO and Sirt3^fl/fl^ mouse models. We found that, compared with control littermates, the inhibitory effect of P7C3-A20 on microglia-mediated neuroinflammation was partially suppressed in Sirt3 CKO mice. Specifically, P7C3-A20 significantly reduced the level of activated microglia compared with the ICH group in Sirt3^fl/fl^ mice, while we did not observe the same protective effect of P7C3-A20 compared with the ICH group in Sirt3 CKO mice (Figure 6(a)). Meanwhile, P7C3-A20 significantly suppressed the expression of Nfkb2, Nlrp3, IL-1b, and Ccl2 at the transcriptional level compared to ICH group in Sirt3^fl/fl^ mice, but not in Sirt3 CKO mice (Nfkb2, *F* [2, 15] = 48.89, Sirt3^fl/fl^: ICH vs. ICH+P7C3-A20, *p* = 0.0067, Sirt3^CKO^: ICH vs. ICH+P7C3-A20 *p* = 0.2014; Nlrp3, *F* [2, 15] = 42.48, Sirt3^fl/fl^: ICH vs. ICH+P7C3-A20, *p* = 0.0016, Sirt3^CKO^: ICH vs. ICH+P7C3-A20, *p* = 0.253; IL-1b, F [2, 15]=474.6, Sirt3^fl/fl^: ICH vs. ICH+P7C3-A20 *p* = 0.0014, Sirt3^CKO^: ICH vs. ICH+P7C3-A20, *p* = 0.1978; Ccl2, *F* [2, 15] = 154.5, Sirt3^fl/fl^: ICH vs. ICH+P7C3-A20 *p* < 0.0001, Sirt3^CKO^: ICH vs. ICH+P7C3-A20, *p* = 0.1051) (Figures 6(b)–6(e)). All these results indicated that Sirt3 CKO in the microglia countered the inhibitory effect of P7C3-A20 on neuroinflammation in mice.

### 3.7. Microglial Sirt3 CKO Countered the Protective Effects of P7C3-A20 on BBB, Brain Water Content, and Neurological Function in ICH Mice

**Figure 7:**
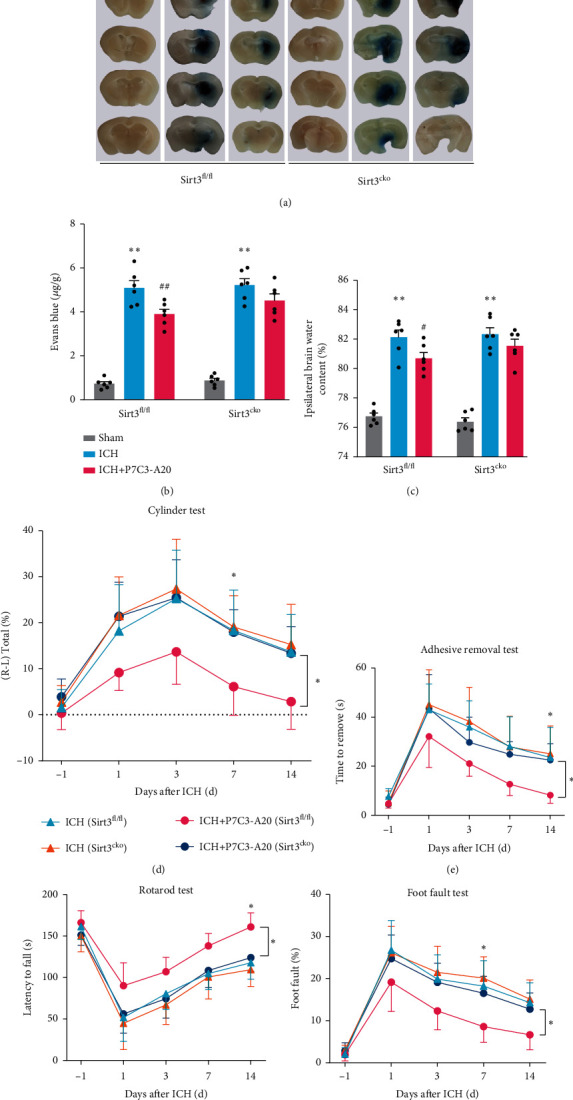
Microglial Sirt3 knockout countered the protective effects of P7C3-A20 on BBB impairment, brain water content, and neurological function scores in mice. (a, b) Representative brain sections with Evans blue and its quantification from Sirt3^fl/fl^ and Sirt3 CKO mice after different treatments, respectively. Sirt3^fl/fl^: ^∗∗^*P* < 0.01, ICH vs. sham; ^##^*P* < 0.01, ICH vs. ICH+P7C3-A20; *n* = 6; Sirt3^CKO^: ^∗∗^*P* < 0.01, ICH vs. sham; *n* = 6. (c) Brain edema statistical analysis in Sirt3^fl/fl^ and Sirt3 CKO mice after different treatments. Sirt3^fl/fl^: ^∗∗^*P* < 0.01, ICH vs. sham; ^##^*P* < 0.01, ICH vs. ICH+P7C3-A20; *n* = 6; Sirt3^CKO^: ^∗∗^*P* < 0.01, ICH vs. sham, *n* = 6. (d) Cylinder test, (e) adhesive removal test, (f) latency to fall test, and (g) grid-walking test were used to assess neurological recovery from Sirt3^fl/fl^ and Sirt3 CKO mice after different treatments. ^∗^*P* < 0.05, ICH+P7C3-A20 (Sirt3^fl/fl^) vs. ICH+P7C3-A20 (Sirt3^CKO^); *n* = 6. The asterisks at the right indicate the group differences. The asterisks on the top indicate single day comparison between the two groups. Values are expressed as mean ± SD. Significance was determined by two-way repeated ANOVA with Bonferroni post hoc tests (b, c, d, e, f, and g).

To confirm the role of Sirt3 in the protective effects of P7C3-A20 on BBB, brain water content, and neurological function, we explored those indexes in both Sirt3 CKO and Sirt3^fl/fl^ mouse models. In line with the above results, we found the inhibitory effect of P7C3-A20 on BBB impairment measured by Evans blue extravasation was also partially suppressed in Sirt3 CKO mice compared with that in Sirt3^fl/fl^ mice (*F* [2, 15] = 175, Sirt3^fl/fl^: ICH vs. ICH+P7C3-A20, *p* = 0.0038, Sirt3^CKO^: ICH vs. ICH+P7C3-A20 *p* = 0.1296) (Figures 7(a) and 7(b)). We also found the same evidence in brain water content that P7C3-A20 failed to alleviate ICH-induced brain edema in Sirt3 CKO mice, but could alleviate the brain edema in Sirt3^fl/fl^ mice (*F* [2, 15] = 104.7, Sirt3^fl/fl^: ICH vs. ICH+P7C3-A20, *p* = 0.0439, Sirt3^CKO^: ICH vs. ICH+P7C3-A20 *p* = 0.4851) (Figure 7(c)). Then, we measured whether Sirt3 CKO countered the protective effects of P7C3-A20 on the post-ICH sensorimotor function. As expected, we did not observe apparent inhibitory effects of P7C3-A20 on ICH-induced neurological deficits (measured by cylinder test (Figure 7(d)), adhesive removal test (Figure 7(e)), latency to fall test (Figure 7(f)), and grid-walking test (Figure 7(g)) in Sirt3 CKO mice, compared to those protective effects in Sirt3^fl/fl^ mice (cylinder test, *F* [2, 15] = 4.352 Sirt3^fl/fl^+ICH+P7C3-A20 vs. Sirt3^CKO^+ICH+P7C3-A20 *p* = 0.0491; adhesive removal, *F* [2, 15] = 7.121 Sirt3^fl/fl^+ICH+P7C3-A20 vs. Sirt3^CKO^+ICH+P7C3-A20 *p* = 0.0362; latency to fall test, *F* [2, 15] = 5.597 Sirt3^fl/fl^+ICH+P7C3-A20 vs. Sirt3^CKO^+ICH+P7C3-A20 *p* = 0.032; grid-walking test, *F* [2, 15] = 6.376, Sirt3^fl/fl^+ICH+P7C3-A20 vs. Sirt3^CKO^+ICH+P7C3-A20 *p* = 0.0495). All these results indicated that microglial Sirt3 knockout countered the protective effects of P7C3-A20 for neurological recovery after ICH.

### 3.8. Plasma NAD^+^ Levels in ICH Patients Correlate with Long-Term Outcome

**Figure 8:**
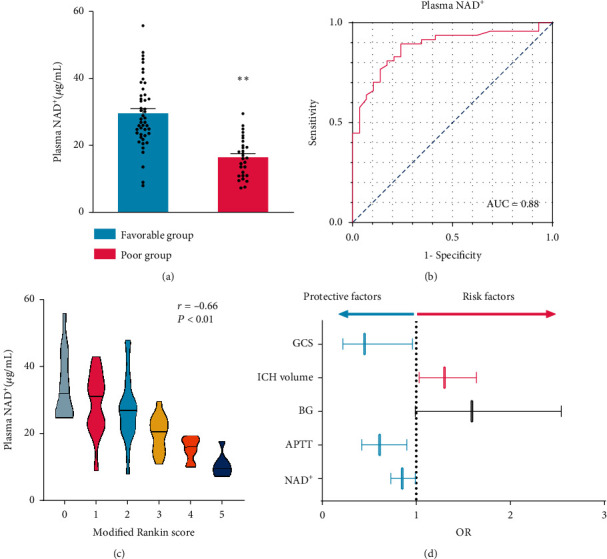
Plasma NAD^+^ levels in patients with ICH correlate with long-term outcomes. (a) Plasma NAD^+^ level indicating a significant decrease in patients with poor outcomes. ^∗∗^*P* < 0.01, favorable group vs poor group. (b) ROC analysis of the discriminative ability of plasma NAD^+^ levels for risk of poor outcome in patients with ICH. Area under the curve 0.88, optimal cut-off point 20.3 *μ*g/mL, sensitivity 89.4%; specificity 75.9%. (C) Linear correlation between plasma NAD^+^ levels and long-term neurological outcome assessed by mRS scores (*r* = −0.66 and *P* < 0.01). (d) Forest plot of multivariable regression analyses. Candidate variables for inclusion included GCS, ICH volume, BG, APTT, and NAD^+^ (*P* < 0.05 for all comparisons except for BG). GCS: Glasgow Coma Scale; BG: blood glucose; APTT: activated partial thromboplastin time; OR: odds ratio.

**Figure 9:**
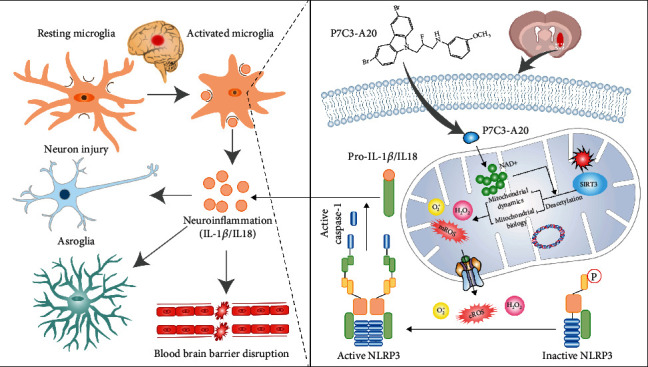
The proposed mechanism of P7C3-A20 induced mitochondrial dynamics and biology alterations in microglia. P7C3-A20 could increase mitochondrial NAD^+^ level, which blocked excessive production of superoxide, maintained mitochondrial quality control, and inhibited microglia-mediated neuroinflammation in a Sirt3-dependent manner.

To identify the translational relevance of our data, the plasma NAD^+^ levels in ICH patients were examined. A total of 76 survived ICH patients managed nonoperatively were included in the study. Patients with favorable outcomes had a significantly higher plasma NAD^+^ level than those with poor outcomes (*t* [73.84] = 7.202, *p* < 0.0001) (Figure 8(a)). Receiver operating curve (ROC) analysis was performed to evaluate the discriminative ability of plasma NAD^+^ level. A cut-off value of 20.3 *μ*g/mL of NAD^+^ showed the strongest discriminative ability (sensitivity 89.4%; specificity 75.9%; Figure 8(b)). To determine the correlation between the plasma NAD^+^ level and the outcome assessed by 3-month mRS score, the Spearman correlation coefficient was carried out, indicating a positive correlation between them (*r* [74] = −0.66 and *p* < 0.001; Figure 8(c)). After adjusting for Glasgow Coma Scale (GCS) and other critical outcome-associated factors at presentation, there was a significant positive relationship between adjusted long-term outcome and plasma NAD^+^ level (Figure 8(d)).  Table 1 shows the baseline clinical characteristics of the study cohort. A high plasma NAD^+^ level, a continuous variable, was a significant independent predictor of favorable outcomes in both univariate and multivariate logistic analyses (OR = 0.85, 95% CI 0.73-0.99, adjusted *p* = 0.033; Table 1). All clinical results indicated a positive correlation between the plasma NAD^+^ level and the good outcome in ICH patients. At last, a diagram of the molecular mechanism elucidated in this study is shown in Figure 9.

## 4. Discussion

This study is aimed at exploring the therapeutic potential of P7C3-A20 in ICH-induced brain injury and its molecular mechanisms. P7C3-A20-treated mice showed an evident decrease in lesion volume, BBB impairment, brain edema, and neural apoptosis after ICH, as well as a significant improvement in sensorimotor ability. Additionally, RNA sequencing and subsequent experiments revealed that P7C3-A20 treatment markedly suppressed microglia-mediated neuroinflammation. Importantly, we used models of microglia-specific Sirt3 CKO mice and Sirt3 knockout BV2 cells to elucidate that P7C3-A20 exerted protective functions against neuroinflammation and mitochondrial damage in a Sirt3-dependent manner. Specifically, P7C3-A20 treatment enhanced NAD^+^ level, promoted Sirt3-deacetylation activities, improved mitochondrial dynamics and mitochondrial bioenergetics, and blocked inflammatory response activation. Finally, we documented a correlation between plasma NAD^+^ levels in ICH patients and neurological outcomes. Taken together, we propose that P7C3-A20 is a promising therapeutic agent for neuroinflammatory injury after ICH and functions, at least partly, through activating the Sirt3 pathway.

NAD^+^ is an essential enzyme that involves a broad number of cellular functions and has a vital role in maintaining organism homeostasis [10]. It not only acts as a coenzyme in reduction-oxidation reactions and regulator of cellular metabolism and mitochondrial respiration but also a critical cosubstrate for additional pleiotropic enzymes [40]. NAD^+^ depletion usually occurs in acute brain injury [11, 13, 14]. Reduced NAD^+^ level was found after TBI, which depleted ATP, caused cellular energy failure, and initiated mitochondria-mediated apoptosis [41]. Pieper et al. also reported that NAD^+^ depletion occurred within 30 minutes after ischemia, leading to initial DNA damage. Consistent with previous findings, our study found that the plasma NAD^+^ level in the ICH patients with favorable outcomes was higher than that in patients with poor outcomes. This finding might indicate that the patients who suffered from more severe ICH were likely to consume more NAD^+^. In addition, we demonstrated a positive correlation between a higher plasma NAD^+^ level and a better outcome in ICH patients, all suggesting that NAD^+^ is a regulator of ICH outcomes and NAD^+^ supplementation may be an effective therapy protecting against post-ICH brain injury.

P7C3-A20, one of the active derivatives of P7C3, has been designed with better proneurogenic activity than P7C3 [42]. P7C3-A20 was shown to improve NAD^+^ level through the NAMPT-mediated NAD^+^ biosynthesis salvage pathway, in which P7C3-A20 could bind to NAMPT and enhance the activity of purified NAMPT enzyme [15]. Importantly, P7C3-A20 is safe with the ability to cross the blood-brain barrier [42]. Increasing reports have demonstrated that P7C3-A20 exerted neuroprotective and proneurogenic effects in several animal models of human diseases, such as traumatic brain injury, ischemic stroke, and neurodegenerative diseases [16, 17, 43]. However, the effect of P7C3-A20 on ICH has never been explored. In the present study, we found that P7C3-A20 administration significantly decreased lesion volume, BBB disruption, brain edema, and neuronal necrosis at 3 d after ICH. Using the neurobehavioral test to evaluate sensorimotor capacity, P7C3-A20-treated mice showed stronger sensorimotor ability than ICH mice at 1, 3, 7, and 14 d after ICH. These results suggested that P7C3-A20 could alleviate post-ICH secondary brain injury to improve sensorimotor function.

Neuroinflammation is a critical link in secondary brain injury after ICH [4]. Within minutes to hours after the ictus of ICH, neuroinflammation occurs, featuring activation of glia cells, and the release of cytotoxic factors, reactive oxygen species, matrix metalloproteinases, etc. [4, 44]. Over time, neuroinflammation induces aggravation and persistence of secondary brain injury, which is the main culprit of neurological deficits. In our study, RNA sequencing revealed that ICH-induced neuroinflammation was significantly suppressed after P7C3-A20 treatment, indicating that P7C3-A20 possessed anti-inflammatory properties. Microglia, the dominant immune cell in the central nervous system, participates in the activation and regulation of neuroinflammation after ICH [45]. Once activated, microglia release proinflammatory factors and cytotoxic substances to disrupt the blood-brain barrier (BBB), thus leading to neuronal damage [46]. As this process may determine the destiny of neuroinflammation, microglia-mediated neuroinflammation has been targeted in various therapeutic interventions, but only to show limited efficacies [4]. In this study, we found that P7C3-A20 efficiently reduced the level of CD68, reversed microglia morphological change, and suppressed microglia-mediated neuroinflammation after ICH, indicating its capability to stabilize microglia in the resting phenotype under ICH conditions.

Maintenance of mitochondrial quality control is essential for cells to function normally [47]. When the mitochondria are damaged, excessive ROS may be released from mitochondria and lead to cellular damage [48]. For instance, excess ROS can directly trigger cell lipid peroxidation and disrupt DNA integrity after ICH, thereby activating inflammatory cells and exacerbating inflammation, especially microglia and NLRP3 [49]. After ICH, ROS overaccumulate in patients and animal models and has been proven to be associated with secondary brain injury [4]. Thus, mitochondria quality control has been identified as a potential target in modulating neuroinflammation for neurological disorders. NAD^+^ is indispensable for many vital enzymes in mitochondria and is implicated in mitochondria quality control [10, 40, 50]. During stroke, the regulation of NAD^+^ level could affect mitochondrial function and ROS production [51]. In line with previous studies, our study revealed that P7C3-A20 significantly enhanced the flux of NAD^+^, suppressed mitochondrial ROS production, and attenuated the impairment of mitochondrial dynamics and bioenergetic system after OxyHb treatment. These results indicated that P7C3-A20-mediated enhancement of NAD^+^ maintained mitochondrial quality after ICH.

How P7C3-A20 modulated mitochondrial quality control was unclear. NAD^+^ functions are primarily mediated by interactions with a group of NAD^+^-dependent proteins, such as the sirtuin family [23]. Among the sirtuin family, Sirt3, the most abundant deacetylase in mitochondria, is a central regulatory hub for mitochondrial function and energy metabolism [20]. Recent evidence showed that reduced deacetylase activity of Sirt3 may link mitochondrial dysfunction with the initiation of the inflammatory response during stroke [51]. Zheng et al. reported that Sirt3-deacetylase activity decreased to the minimum at 24 h after ICH, and Sirt3 activation could ameliorate oxidative stress and mitochondrial dysfunction in diabetic rats [21]. Combining the above roles played by Sirt3-related mitochondrial quality control in neuroprotection, it is very worthwhile to further explore whether P7C3-A20 relies on Sirt3 to protect against ICH-induced injury. Consistent with the above results, we found that in the ICH models of BV2 cells and Sirt3^fl/fl^ mice, P7C3-A20 blocked excessive superoxide production, maintained mitochondrial quality, and inhibited microglia-mediated neuroinflammation, all combined to attenuate brain injuries and neurological deficits. Conversely, these protective effects were discounted after the Sirt3 knockout in vitro, and similar effects were also found in the Sirt3 CKO mice. These results suggested that P7C3-A20 inhibited microglial inflammation and attenuated mitochondrial impairment after ICH, at least partly, via the NAD^+^-Sirt3 axis.

Several limitations should be noted. First, we only verified the protective effect of P7C3-A20 against post-ICH early-stage brain injury; the effect in the late stage is not yet clear. Second, the young healthy animals used in our experiment were not submitted to other medications. However, ICH patients, always complicated with severe comorbidities (diabetes, cardiovascular diseases, etc.), are often pretreated with drugs. In this way, their use should be considered in further explorations.

## 5. Conclusions

In summary, the present study demonstrates that P7C3-A20 is a promising therapeutic agent for brain injury after ICH. Meanwhile, we also discovered that P7C3-A20 protects against microglial activation and inflammatory response through activating Sirt3-mediated mitochondrial quality control.

## Figures and Tables

**Table 1:** Characteristics of the study patients.

Variables	Study population (*n* = 76)	Favorable outcome (*n* = 47)	Poor outcome (*n* = 29)	Crude OR (95% CI)	Crude *p* value	Adjusted OR (95% CI)	Adjusted *p* value
*Demographics (age,mean* ± *SD; male, %)*							
Age (*y*)	60.13 (13.36)	59.28 (13.95)	61.76 (13.00)	0.98-1.05	0.437		
Male	44 (57.9)	30 (63.8)	14 (48.3)	0.21-1.35	0.184		
*Vital signs (mean* ± *SD*)							
Systolic BP (mmHg)	154.35 (26.36)	150.17 (25.02)	161.14 (27.51)	1-1.03	0.082		
Diastolic BP (mmHg)	87.38 (13.92)	86.34 (12.76)	89.07 (15.72)	0.98-1.05	0.405		
Pulse rate (beats/min)	75.60 (11.81)	74.94 (11.70)	76.69 (12.12)	0.97-1.05	0.528		
Breathing rate (breaths/min)	19.07 (2.46)	19.09 (1.50)	19.07 (3.55)	0.83-1.2	0.978		
*Clinical characteristics (mean* ± *SD*)							
GCS	12.51 (2.61)	13.87 (1.06)	10.31 (2.88)	0.2-0.56	<0.001	0.22-0.96	0.039
ICH volume (mL)	23.64 (8.02)	19.57 (6.16)	30.28 (6.05)	1.15-1.43	<0.001	1.03-1.64	0.026
*Laboratory test (mean* ± *SD*)							
WBC (10^9^/L)	9.33 (3.70)	8.77 (3.68)	10.26 (3.62)	0.98-1.27	0.097		
AST/ALT	1.43 (0.90)	1.53 (1.06)	1.29 (0.54)	0.37-1.34	0.281		
Scr (*μ*mol/L)	80.76 (105.35)	82.83 (122.15)	77.43 (72.09)	0.99-1	0.828		
BUN (*μ*mol/L)	6.30 (5.68)	5.57 (3.69)	7.50 (7.85)	0.97-1.18	0.198		
GFR (mL/min)	94.67 (23.98)	94.01 (23.11)	95.73 (25.72)	0.98-1.02	0.76		
BG (mmol/L)	7.80 (3.33)	6.67 (1.65)	9.66 (4.41)	1.16-1.88	0.002	0.99-2.54	0.56
APTT (*s*)	25.50 (4.48)	26.55 (3.90)	23.80 (4.90)	0.74-0.96	0.012	0.42-0.9	0.013
FDP (mg/L)	11.10 (29.17)	7.85 (15.09)	16.38 (43.13)	0.99-1.03	0.282		
*NAD+ (μg/mL)*	24.67 (10.80)	29.71 (10.08)	16.53 (5.88)	0.73-0.89	<0.001	0.73-0.99	0.033

Abbreviations: BP: blood pressure; GCS: Glasgow Coma Scale; WBC: white blood cells; AST/ALT: aspartate aminotransferase to alanine aminotransferase ratio; Scr: serum creatinine; BUN: blood urea nitrogen; GFR: glomerular filtration rate; BG: blood glucose; APTT: activated partial thromboplastin time; FDP: fibrin degradation products.

## Data Availability

The data presented in this study are available on reasonable request from the corresponding author.

## Abbreviations

ICH: Intracerebral hemorrhage
NAD^+^: Nicotinamide adenine dinucleotide
BBB: Blood-brain barrier
ROS: Reactive oxygen species
PBS: Phosphate buffered saline
mRS: Modified Rankin scale
OxyHb: Oxyhemoglobin
MMP: Mitochondrial membrane potential
NAMPT: Nicotinamide phosphoribosyl transferase
CKO: Conditional knockouts
ROC: Receiver operating curve
GCS: Glasgow Coma Scale.

## Supplementary Materials

Supplementary 1 Supplementary Table 1: primers for RT-PCR.

Supplementary 2 Supplementary Table 2: statistical table.

Supplementary 3 Supplementary Figure 1: GO enrichment analysis of the overlapped genes in the Venn diagram.

Supplementary 4 Supplementary Figure 2: the neuroprotective effects of the different doses of P7C3-A20 on neuroinflammatory injury after ICH. (A) Immunofluorescence staining for Iba1 (red) with CD68 (green) revealing the activated microglia levels in different dose groups. Each micrograph next to the boxed area shows an amplified version of the white square in merged images. Scale bar, 50 *μ*m. (B) Statistical analysis of activated microglia number. ^∗∗∗^*P* < 0.001, ns. *P* > 0.05, and *n* = 6. (c) Analysis of the quantitative fluorescence intensity for CD68. ^∗^*P* < 0.05, ^∗∗∗^*P* < 0.001, and *n* = 6. Values are expressed as mean ± SD. Significance was determined by one-way ANOVA with Bonferroni post hoc tests (B, C).

Supplementary 5 Supplementary Figure 3: qPCR was used to validate the trend in the expression of microglial activation marker at the transcriptional level. (A) CD11b mRNA level. ^∗∗∗^*P* < 0.001 and *n* = 6. (B) IONS mRNA level. ^∗∗∗^*P* < 0.001 and *n* = 6. (C) CD206 mRNA level. ^∗^*P* < 0.05, ^∗∗^*P* < 0.01, and *n* = 6. (D) Arg1 mRNA level. ^∗∗∗^*P* < 0.001 and *n* = 6. As shown in A-D, the ICH group markedly increased CD11b and INOS and decreased CD206 and Arg1 at the transcriptional level compared to the sham, while those trends were reversed by additional P7C3-A20 treatment. Values are expressed as mean ± SD. Significance was determined by one-way ANOVA with Bonferroni post hoc tests (A, B, C, and D).

Supplementary 6 Supplementary Figure 4: P7C3-A20 showed no effect on the OxyHb-induced mitochondrial autophagy process. (A-D) Representative Western blot images of Parkin, PINK1, and Tom20 and the related statistical analysis. Western blotting displayed an evident fall of Tom20 and a rise of Parkin and PINK1 in the ICH group compared to the vehicle, while those changes were not reversed by additional P7C3-A20 treatment. Notably, there was no statistical significance between ICH, ICH+P7C3-A20, and CCCP (positive control) groups. ^∗∗∗^*P* < 0.001, ICH vs. sham. Values are expressed as mean ± SD. Significance was determined by one-way ANOVA with Bonferroni post hoc tests (B, C, and D).
