# Supplementary material for: P7C3-A20 Attenuates Microglial Inflammation and Brain Injury after ICH through Activating the NAD+/Sirt3 Pathway
Source: Oxid Med Cell Longev. 2023 Feb 8;2023:7857760. doi: 10.1155/2023/7857760 (PMC9936507; doi:10.1155/2023/7857760)
Supplement: Supplementary 1 — Supplementary Table 1: primers for RT-PCR. [file 7857760.f1.pdf]

**Supplementary Table 1. Primers for RT-PCR.**

| Primers | | Sequence |
|---|---|---|
| NLRP3 | Forward | ATT ACC CGC CCG AGA AAG G |
| | Reverse | TCG CAG CAA AGA TCC ACA CAG |
| Nfkb2 | Forward | TGGAACAGCCCAAACAGC |
| | Reverse | CACCTGGCAAACCTCCAT |
| Ccl2 | Forward | TCCACGCCAATTCATCGTT |
| | Reverse | TCCCAGCCAGGTGTCATTTT |
| IL1b | Forward | CCAGAGATACAAAGAAATGATGG |
| | Reverse | ACTCCAGAAGACCAGAGGAAAT |
| CD11b | Forward | ACAGACAAACAGCCCAAACC |
| | Reverse | GCCTCACCCATCAGTTGTTT |
| CD206 | Forward | CTCTGTTCAGCTATTGGACGC |
| | Reverse | CGGAATTTCTGGGATTCAGCTTC |
| INOS | Forward | ATGGACCAGTATAAGGCAAGC |
| | Reverse | GCTCTGGATGAGCCTATATTG |
| Arg1 | Forward | CTCCAAGCCAAAGTCCTTAGAG |
| | Reverse | AGGAGCTGTCATTAGGGACATC |
| β-actin | Forward | GTG CTA TGT TGC TCT AGA CTT CG |
| | Reverse | ATG CCA CAG GAT TCC ATA CC |
