# Supplementary material for: P7C3-A20 Attenuates Microglial Inflammation and Brain Injury after ICH through Activating the NAD+/Sirt3 Pathway
Source: Oxid Med Cell Longev. 2023 Feb 8;2023:7857760. doi: 10.1155/2023/7857760 (PMC9936507; doi:10.1155/2023/7857760)
Supplement: Supplementary 2 — Supplementary Table 2: statistical table. [file 7857760.f2.pdf]

Supplementary Table 2. Statistical Table

Figure1	n	Data structure	Test used	Statistic	P value
1B	(-1, 1, 3, 7, 14d):<br>Sham=6<br>ICH=6<br>ICH+P7C3-A20(5mg/kg) =6<br>ICH+P7C3-A20(10mg/kg) =6<br>ICH+P7C3-A20(20mg/kg) =6	Normal distribution	2-way repeated ANOVA,<br>Bonferroni post hoc	F(4, 25)=22.84	P<0.0001<br>Sham vs ICH p<0.0001<br>ICH vs ICH+P7C3-A20(5mg/kg) p=0.4076<br>ICH vs ICH+P7C3-A20(10mg/kg) p=0.0436<br>ICH vs ICH+P7C3-A20(20mg/kg) p=0.0225<br>D3: ICH vs ICH+P7C3-A20(20mg/kg) p=0.0263<br>D7: ICH vs ICH+P7C3-A20(10mg/kg) p=0.0230<br>ICH vs ICH+P7C3-A20(20mg/kg) p=0.0101<br>D14: ICH vs ICH+P7C3-A20(10mg/kg) p=0.0347
1C	(-1, 1, 3, 7, 14d):<br>Sham=6<br>ICH=6<br>ICH+P7C3-A20(5mg/kg) =6<br>ICH+P7C3-A20(10mg/kg) =6<br>ICH+P7C3-A20(20mg/kg) =6	Normal distribution	2-way repeated ANOVA,<br>Bonferroni post hoc	F(4, 25)=23.81	P<0.0001<br>Sham vs ICH p<0.0001<br>ICH vs ICH+P7C3-A20(5mg/kg) p=0.15651<br>ICH vs ICH+P7C3-A20(10mg/kg) p=0.0418<br>ICH vs ICH+P7C3-A20(20mg/kg) p=0.0212
1D	(-1, 1, 3, 7, 14d):<br>Sham=6<br>ICH=6<br>ICH+P7C3-A20(5mg/kg) =6<br>ICH+P7C3-A20(10mg/kg) =6<br>ICH+P7C3-A20(20mg/kg) =6	Non-normal distribution	2-way repeated ANOVA,<br>Bonferroni post hoc	F(4, 25)=18.07	P<0.0001<br>Sham vs ICH p<0.0001<br>ICH vs ICH+P7C3-A20(5mg/kg) p=0.6048<br>ICH vs ICH+P7C3-A20(10mg/kg) p=0.0135<br>ICH vs ICH+P7C3-A20(20mg/kg) p=0.0115<br>D3: ICH vs ICH+P7C3-A20(20mg/kg) p=0.0222<br>D7: ICH vs ICH+P7C3-A20(10mg/kg) p=0.0178<br>ICH vs ICH+P7C3-A20(20mg/kg) p=0.0451<br>D14: ICH vs ICH+P7C3-A20(10mg/kg) p=0.0322<br>ICH vs ICH+P7C3-A20(20mg/kg)

					p=0.0311
1E	(-1, 1, 3, 7, 14d):<br>Sham=6<br>ICH=6<br>ICH+P7C3-A20(5mg/kg) =6<br>ICH+P7C3-A20(10mg/kg) =6<br>ICH+P7C3-A20(20mg/kg) =6	Normal distribution	2-way repeated ANOVA,<br>Bonferroni post hoc	F(4, 25)=26.11	P<0.0001<br>Sham vs ICH p<0.0001<br>ICH vs ICH+P7C3-A20(5mg/kg) p=0.5571<br>ICH vs ICH+P7C3-A20(10mg/kg) p=0.0108<br>ICH vs ICH+P7C3-A20(20mg/kg) p=0.0368<br>D7: ICH vs ICH+P7C3-A20(20mg/kg) p=0.0306<br>D14: ICH vs ICH+P7C3-A20(10mg/kg) p=0.0363
1G	ICH=6<br>ICH+P7C3-A20=6	Normal distribution	t test	t[10]=2.437	P=0.0329
1H	(Ipsilateral, Contralateral, Cerebellum):<br>Sham=6<br>ICH=6<br>ICH+P7C3-A20=6	Normal distribution	2-way repeated ANOVA,<br>Bonferroni post hoc	F(2, 15)=28.74	P<0.0001<br>Ipsilateral: Sham vs ICH p=0.0005<br>Sham vs ICH+P7C3-A20 p=0.0001<br>ICH vs ICH+P7C3-A20 p=0.0415
Figure2	n	Data structure	Test used	Statistic	P value
2B	Sham=6<br>ICH=6<br>ICH+P7C3-A20=6	Normal distribution	1-way ANOVA,<br>Bonferroni post hoc	F(2, 15)=69.01	P<0.0001<br>Sham vs ICH p<0.0001<br>Sham vs ICH+P7C3-A20 p<0.0001<br>ICH vs ICH+P7C3-A20 p=0.0155
2D	Sham=6<br>ICH=6<br>ICH+P7C3-A20=6	Normal distribution	1-way ANOVA,<br>Bonferroni post hoc	F(2, 15)=16.55	P=0.0002<br>Sham vs ICH p=0.0001<br>Sham vs ICH+P7C3-A20 p=0.1055<br>ICH vs ICH+P7C3-A20 p=0.0118
2E	Sham=6<br>ICH=6<br>ICH+P7C3-A20=6	Normal distribution	1-way ANOVA,<br>Bonferroni post hoc	F(2, 15)=14.70	P=0.0003<br>Sham vs ICH p=0.0004<br>Sham vs ICH+P7C3-A20 p>0.9999<br>ICH vs ICH+P7C3-A20 p=0.0025
2F	Sham=6<br>ICH=6<br>ICH+P7C3-A20=6	Normal distribution	1-way ANOVA,<br>Bonferroni post hoc	F(2, 15)=39.38	P<0.0001<br>Sham vs ICH p<0.0001<br>Sham vs ICH+P7C3-A20 p=0.0003<br>ICH vs ICH+P7C3-A20 p=0.0067
2H	Sham=6<br>ICH=6<br>ICH+P7C3-A20=6	Normal distribution	1-way ANOVA,<br>Bonferroni post hoc	F(2, 15)=73.49	P<0.0001<br>Sham vs ICH p<0.0001<br>Sham vs ICH+P7C3-A20 p<0.0001<br>ICH vs ICH+P7C3-A20 p<0.0001
2J	Sham=6<br>ICH=6	Normal distribution	1-way ANOVA,<br>Bonferroni post hoc	F(2, 15)=90.40	P<0.0001<br>Sham vs ICH p<0.0001

	n	Data structure	Test used	Statistic	P value
	ICH+P7C3-A20=6				Sham vs ICH+P7C3-A20 p<0.0001<br>ICH vs ICH+P7C3-A20 p<0.0001
2K	Sham=6<br>ICH=6<br>ICH+P7C3-A20=6	Normal distribution	1-way ANOVA, Bonferroni post hoc	F(2, 15)=33.58	P<0.0001<br>Sham vs ICH p<0.0001<br>Sham vs ICH+P7C3-A20 p=0.0006<br>ICH vs ICH+P7C3-A20 p=0.0153
Figure3	n	Data structure	Test used	Statistic	P value
3E	Sham=6<br>ICH=6<br>ICH+P7C3-A20=6	Normal distribution	1-way ANOVA, Bonferroni post hoc	F(2, 15)=30.41	P<0.0001<br>Sham vs ICH p<0.0001<br>Sham vs ICH+P7C3-A20 p=0.0099<br>ICH vs ICH+P7C3-A20 p=0.0019
3F	Sham=6<br>ICH=6<br>ICH+P7C3-A20=6	Normal distribution	1-way ANOVA, Bonferroni post hoc	F(2, 15)=45.35	P<0.0001<br>Sham vs ICH p<0.0001<br>Sham vs ICH+P7C3-A20 p=0.4617<br>ICH vs ICH+P7C3-A20 p<0.0001
3G	Sham=6<br>ICH=6<br>ICH+P7C3-A20=6	Non-normal distribution	1-way ANOVA, Bonferroni post hoc	F(2, 15)=94.06	P<0.0001<br>Sham vs ICH p<0.0001<br>Sham vs ICH+P7C3-A20 p<0.0001<br>ICH vs ICH+P7C3-A20 p=0.0079
3H	Sham=6<br>ICH=6<br>ICH+P7C3-A20=6	Normal distribution	1-way ANOVA with Welch's correction	W(2, 6.899)=234.4	P<0.0001<br>Sham vs ICH p<0.0001<br>Sham vs ICH+P7C3-A20 p<0.0001<br>ICH vs ICH+P7C3-A20 p=0.0026
3K	Sham=20<br>ICH=20<br>ICH+P7C3-A20=20	Normal distribution	1-way ANOVA, Bonferroni post hoc	F(2, 15)=6.077	P=0.0041<br>Sham vs ICH p=0.0058<br>Sham vs ICH+P7C3-A20 p>0.9999<br>ICH vs ICH+P7C3-A20 p=0.0262
3L	Sham=20<br>ICH=20<br>ICH+P7C3-A20=20	Normal distribution	1-way ANOVA, Bonferroni post hoc	F(2, 57)=10.72	P=0.0001<br>Sham vs ICH p<0.0001<br>Sham vs ICH+P7C3-A20 p=0.1542<br>ICH vs ICH+P7C3-A20 p=0.0332
3M	Sham=20<br>ICH=20<br>ICH+P7C3-A20=20	Non-normal distribution	1-way ANOVA, Bonferroni post hoc	F(2, 57)=14.85	P<0.0001<br>Sham vs ICH p<0.0001<br>Sham vs ICH+P7C3-A20 p=0.2121<br>ICH vs ICH+P7C3-A20 p=0.0026
3N	Sham=20<br>ICH=20<br>ICH+P7C3-A20=20	Normal distribution	1-way ANOVA with Welch's correction	W(2, 35.09)=58.05	P<0.0001<br>Sham vs ICH p<0.0001<br>Sham vs ICH+P7C3-A20 p=0.0546<br>ICH vs ICH+P7C3-A20 p<0.0001
Figure4	n	Data structure	Test used	Statistic	P value
4B	Con: Vehicle=6<br>ICH=6<br>ICH+P7C3-A20=6<br>Sirt3 KO: Vehicle=6	Normal distribution	2-way repeated ANOVA, Bonferroni post hoc	F(2, 15)=358.8	P<0.0001<br>Con: Vehicle vs OxyHb p<0.0001<br>Vehicle vs OxyHb+P7C3-A20 p<0.0001<br>ICH vs OxyHb+P7C3-A20 p<0.0001

	ICH=6<br>ICH+P7C3-A20=6				Sirt3 KO: Vehicle vs OxyHb p<0.0001<br>Vehicle vs OxyHb+P7C3-A20 p<0.0001<br>OxyHb vs OxyHb+P7C3-A20 p=0.1149
4D	Con: Vehicle=6<br>ICH=6<br>ICH+P7C3-A20=6<br>Sirt3 KO: Vehicle=6<br>ICH=6<br>ICH+P7C3-A20=6	Normal distribution	2-way repeated ANOVA, Bonferroni post hoc	F(2, 15)=49.14	P<0.0001<br>Con: Vehicle vs OxyHb p<0.0001<br>Vehicle vs OxyHb+P7C3-A20 p=0.4062<br>OxyHb vs OxyHb+P7C3-A20 p<0.0001<br>Sirt3 KO: Vehicle vs OxyHb p<0.0001<br>Vehicle vs OxyHb+P7C3-A20 p<0.0001<br>OxyHb vs OxyHb+P7C3-A20 p=0.5408
4F	Con: Vehicle=6<br>ICH=6<br>ICH+P7C3-A20=6<br>Sirt3 KO: Vehicle=6<br>ICH=6<br>ICH+P7C3-A20=6	Normal distribution	2-way repeated ANOVA, Bonferroni post hoc	F(2, 15)=143.8	P<0.0001<br>Con: Vehicle vs OxyHb p<0.0001<br>Vehicle vs OxyHb+P7C3-A20 p=0.0003<br>OxyHb vs OxyHb+P7C3-A20 p<0.0001<br>Sirt3 KO: Vehicle vs OxyHb p<0.0001<br>Vehicle vs OxyHb+P7C3-A20 p<0.0001<br>OxyHb vs OxyHb+P7C3-A20 p=0.6212
4G	Con: Vehicle=6<br>ICH=6<br>ICH+P7C3-A20=6<br>Sirt3 KO: Vehicle=6<br>ICH=6<br>ICH+P7C3-A20=6	Normal distribution	2-way repeated ANOVA, Bonferroni post hoc	F(2, 15)=12.74	P=0.0006<br>Con: Vehicle vs OxyHb p=0.0098<br>Vehicle vs OxyHb+P7C3-A20 p>0.9999<br>OxyHb vs OxyHb+P7C3-A20 p=0.0390<br>Sirt3 KO: Vehicle vs OxyHb p=0.0008<br>Vehicle vs OxyHb+P7C3-A20 p=0.4010<br>OxyHb vs OxyHb+P7C3-A20 p=0.0446
4H	Con: Vehicle=6<br>ICH=6<br>ICH+P7C3-A20=6<br>Sirt3 KO: Vehicle=6<br>ICH=6<br>ICH+P7C3-A20=6	Normal distribution	2-way repeated ANOVA, Bonferroni post hoc	F(2, 15)=78.55	P<0.0001<br>Con: Vehicle vs OxyHb p<0.0001<br>Vehicle vs OxyHb +P7C3-A20 p=0.0013<br>OxyHb vs OxyHb+P7C3-A20 p=0.0035<br>Sirt3 KO: Vehicle vs OxyHb p<0.0001<br>Vehicle vs OxyHb+P7C3-A20 p<0.0001<br>OxyHb vs OxyHb+P7C3-A20 p=0.2849
4I	Con: Vehicle=6<br>ICH=6<br>ICH+P7C3-A20=6<br>Sirt3 KO: Vehicle=6<br>ICH=6<br>ICH+P7C3-A20=6	Normal distribution	2-way repeated ANOVA, Bonferroni post hoc	F(2, 15)=56.50	P<0.0001<br>Con: Vehicle vs ICH p<0.0001<br>Vehicle vs OxyHb+P7C3-A20 p=0.0073<br>OxyHb vs OxyHb+P7C3-A20 p=0.0091<br>Sirt3 KO: Vehicle vs OxyHb p<0.0001<br>Vehicle vs OxyHb+P7C3-A20 p<0.0001<br>OxyHb vs OxyHb+P7C3-A20 p=0.4407
Figure5	n	Data structure	Test used	Statistic	P value
5C	$Sirt3^{fl/fl}$: Sham=6<br>ICH=6<br>ICH+P7C3-A20=6<br>$Sirt3^{CKO}$: Sham=6<br>ICH=6	Normal distribution	2-way repeated ANOVA, Bonferroni post hoc	F(2, 15)=44.82	P<0.0001<br>$Sirt3^{fl/fl}$: Sham vs ICH p<0.0001<br>Sham vs ICH+P7C3-A20 p=0.0005<br>ICH vs ICH+P7C3-A20 p=0.0040<br>$Sirt3^{CKO}$: Sham vs ICH p<0.0001

	ICH+P7C3-A20=6				Sham vs ICH+P7C3-A20 p<0.0001<br>ICH vs ICH+P7C3-A20 p=0.1080
5D	Sirt3$^{fl/fl}$: Sham=6<br>ICH=6<br>ICH+P7C3-A20=6<br>Sirt3^{CKO}: Sham=6<br>ICH=6<br>ICH+P7C3-A20=6	Normal distribution	2-way repeated ANOVA, Bonferroni post hoc	F(2, 15)=124.5	P<0.0001<br>Sirt3$^{fl/fl}$: Sham vs ICH p<0.0001<br>Sham vs ICH+P7C3-A20 p<0.0001<br>ICH vs ICH+P7C3-A20 p<0.0001<br>Sirt3^{CKO}: Sham vs ICH p<0.0001<br>Sham vs ICH+P7C3-A20 p<0.0001<br>ICH vs ICH+P7C3-A20 p=0.0700
5E	Sirt3$^{fl/fl}$: Sham=6<br>ICH=6<br>ICH+P7C3-A20=6<br>Sirt3^{CKO}: Sham=6<br>ICH=6<br>ICH+P7C3-A20=6	Normal distribution	2-way repeated ANOVA, Bonferroni post hoc	F(2, 15)=104.5	P<0.0001<br>Sirt3$^{fl/fl}$: Sham vs ICH p<0.0001<br>Sham vs ICH+P7C3-A20 p<0.0001<br>ICH vs ICH+P7C3-A20 p=0.0419<br>Sirt3^{CKO}: Sham vs ICH p<0.0001<br>Sham vs ICH+P7C3-A20 p<0.0001<br>ICH vs ICH+P7C3-A20 p=0.1963
Figure6	n	Data structure	Test used	Statistic	P value
6B	Sirt3$^{fl/fl}$: Sham=6<br>ICH=6<br>ICH+P7C3-A20=6<br>Sirt3^{CKO}: Sham=6<br>ICH=6<br>ICH+P7C3-A20=6	Normal distribution	2-way repeated ANOVA, Bonferroni post hoc	F(2, 15)=48.89	P<0.0001<br>Sirt3$^{fl/fl}$: Sham vs ICH p<0.0001<br>Sham vs ICH+P7C3-A20 p=0.1471<br>ICH vs ICH+P7C3-A20 p=0.0067<br>Sirt3^{CKO}: Sham vs ICH p<0.0001<br>Sham vs ICH+P7C3-A20 p=0.0011<br>ICH vs ICH+P7C3-A20 p=0.2014
6C	Sirt3$^{fl/fl}$: Sham=6<br>ICH=6<br>ICH+P7C3-A20=6<br>Sirt3^{CKO}: Sham=6<br>ICH=6<br>ICH+P7C3-A20=6	Normal distribution	2-way repeated ANOVA, Bonferroni post hoc	F(2, 15)=42.48	P<0.0001<br>Sirt3$^{fl/fl}$: Sham vs ICH p<0.0001<br>Sham vs ICH+P7C3-A20 p=0.0144<br>ICH vs ICH+P7C3-A20 p=0.0016<br>Sirt3^{CKO}: Sham vs ICH p<0.0001<br>Sham vs ICH+P7C3-A20 p<0.0001<br>ICH vs ICH+P7C3-A20 p=0.2530
6D	Sirt3$^{fl/fl}$: Sham=6<br>ICH=6<br>ICH+P7C3-A20=6<br>Sirt3^{CKO}: Sham=6<br>ICH=6<br>ICH+P7C3-A20=6	Normal distribution	2-way repeated ANOVA, Bonferroni post hoc	F(2, 15)=474.6	P<0.0001<br>Sirt3$^{fl/fl}$: Sham vs ICH p<0.0001<br>Sham vs ICH+P7C3-A20 p<0.0001<br>ICH vs ICH+P7C3-A20 p=0.0014<br>Sirt3^{CKO}: Sham vs ICH p<0.0001<br>Sham vs ICH+P7C3-A20 p<0.0001<br>ICH vs ICH+P7C3-A20 p=0.1978
6E	Sirt3$^{fl/fl}$: Sham=6<br>ICH=6<br>ICH+P7C3-A20=6<br>Sirt3^{CKO}: Sham=6<br>ICH=6<br>ICH+P7C3-A20=6	Normal distribution	2-way repeated ANOVA, Bonferroni post hoc	F(2, 15)=154.5	P<0.0001<br>Sirt3$^{fl/fl}$: Sham vs ICH p<0.0001<br>Sham vs ICH+P7C3-A20 p<0.0001<br>ICH vs ICH+P7C3-A20 p=0.0091<br>Sirt3^{CKO}: Sham vs ICH p<0.0001<br>Sham vs ICH+P7C3-A20 p<0.0001

					ICH vs ICH+P7C3-A20 p=0.1051
Figure7	n	Data structure	Test used	Statistic	P value
7B	Sirt3$^{fl/fl}$: Sham=6<br>ICH=6<br>ICH+P7C3-A20=6<br>Sirt3^{CKO}: Sham=6<br>ICH=6<br>ICH+P7C3-A20=6	Normal distribution	2-way repeated ANOVA, Bonferroni post hoc	F(2, 15)=175.0	P<0.0001<br>Sirt3$^{fl/fl}$: Sham vs ICH p<0.0001<br>Sham vs ICH+P7C3-A20 p<0.0001<br>ICH vs ICH+P7C3-A20 p=0.0038<br>Sirt3^{CKO}: Sham vs ICH p<0.0001<br>Sham vs ICH+P7C3-A20 p<0.0001<br>ICH vs ICH+P7C3-A20 p=0.1296
7C	Sirt3$^{fl/fl}$: Sham=6<br>ICH=6<br>ICH+P7C3-A20=6<br>Sirt3^{CKO}: Sham=6<br>ICH=6<br>ICH+P7C3-A20=6	Normal distribution	2-way repeated ANOVA, Bonferroni post hoc	F(2, 15)=104.7	P<0.0001<br>Sirt3$^{fl/fl}$: Sham vs ICH p<0.0001<br>Sham vs ICH+P7C3-A20 p<0.0001<br>ICH vs ICH+P7C3-A20 p=0.0439<br>Sirt3^{CKO}: Sham vs ICH p<0.0001<br>Sham vs ICH+P7C3-A20 p<0.0001<br>ICH vs ICH+P7C3-A20 p=0.4851
7D	Sirt3$^{fl/fl}$: ICH=6<br>ICH+P7C3-A20=6<br>Sirt3^{CKO}: ICH=6<br>ICH+P7C3-A20=6	Normal distribution	2-way repeated ANOVA, Bonferroni post hoc	F(3, 20)=4.352	P=0.0163<br>Sirt3$^{fl/fl}$+ICH+P7C3-A20 vs Sirt3^{CKO}+ICH+P7C3-A20 p=0.0491<br>D7: Sirt3$^{fl/fl}$+ICH+P7C3-A20 vs Sirt3^{CKO}+ICH+P7C3-A20 p=0.0319
7E	Sirt3$^{fl/fl}$: ICH=6<br>ICH+P7C3-A20=6<br>Sirt3^{CKO}: ICH=6<br>ICH+P7C3-A20=6	Non-normal distribution	2-way repeated ANOVA, Bonferroni post hoc	F(3, 20)=7.121	P=0.0019<br>Sirt3$^{fl/fl}$+ICH+P7C3-A20 vs Sirt3^{CKO}+ICH+P7C3-A20 p=0.0362<br>D14: Sirt3$^{fl/fl}$+ICH+P7C3-A20 vs Sirt3^{CKO}+ICH+P7C3-A20 p=0.0438
7F	Sirt3$^{fl/fl}$: ICH=6<br>ICH+P7C3-A20=6<br>Sirt3^{CKO}: ICH=6<br>ICH+P7C3-A20=6	Normal distribution	2-way repeated ANOVA, Bonferroni post hoc	F(3, 20)=5.597	P=0.0059<br>Sirt3$^{fl/fl}$+ICH+P7C3-A20 vs Sirt3^{CKO}+ICH+P7C3-A20 p=0.032<br>D14: Sirt3$^{fl/fl}$+ICH+P7C3-A20 vs Sirt3^{CKO}+ICH+P7C3-A20 p=0.0438
7G	Sirt3$^{fl/fl}$: ICH=6<br>ICH+P7C3-A20=6<br>Sirt3^{CKO}: ICH=6<br>ICH+P7C3-A20=6	Normal distribution	2-way repeated ANOVA, Bonferroni post hoc	F(3, 20)=6.376	P=0.0033<br>Sirt3$^{fl/fl}$+ICH+P7C3-A20 vs Sirt3^{CKO}+ICH+P7C3-A20 p=0.0495<br>D7: Sirt3$^{fl/fl}$+ICH+P7C3-A20 vs Sirt3^{CKO}+ICH+P7C3-A20 p=0.0295
Figure8	n	Data structure	Test used	Statistic	P value
8A	Favorable group=47<br>Poor group=29	Normal distribution	t test with Welch's correction	t[73.84]=7.202	P<0.0001
8C	N=76	Plasma NAD^+: Normal distribution, MRS: rating scale(0 to 5)	Spearman correlation	R[74] = -0.6581	P<0.0001
Figure S2	n	Data structure	Test used	Statistic	P value

SF2B	Sham=6<br>ICH=6<br>ICH+P7C3-A20 (5mg/kg)=6<br>ICH+P7C3-A20 (10mg/kg)=6<br>ICH+P7C3-A20 (20mg/kg)=6	Normal distribution	1-way ANOVA, Bonferroni post hoc	F(4, 25)=17.22	P<0.0001<br>Sham vs ICH p<0.0001<br>ICH vs ICH+P7C3-A20(5mg/kg) P=0.0595<br>ICH vs ICH+P7C3-A20(10mg/kg) p<0.0001<br>ICH vs ICH+P7C3-A20(20mg/kg) p<0.0001
SF2C	Sham=6<br>ICH=6<br>ICH+P7C3-A20 (5mg/kg)=6<br>ICH+P7C3-A20 (10mg/kg)=6<br>ICH+P7C3-A20 (20mg/kg)=6	Normal distribution	1-way ANOVA, Bonferroni post hoc	F(4, 25)=76.21	P<0.0001<br>Sham vs ICH p<0.0001<br>ICH vs ICH+P7C3-A20(5mg/kg) P=0.0255<br>ICH vs ICH+P7C3-A20(10mg/kg) p<0.0001<br>ICH vs ICH+P7C3-A20(20mg/kg) p<0.0001
Figure S3	n	Data structure	Test used	Statistic	P value
SF3A	Sham=6<br>ICH=6<br>ICH+P7C3-A20=6	Normal distribution	1-way ANOVA, Bonferroni post hoc	F(2, 15)=142.2	P<0.0001<br>Sham vs ICH p<0.0001<br>ICH vs ICH+P7C3-A20 p<0.0001
SF3B	Sham=6<br>ICH=6<br>ICH+P7C3-A20=6	Normal distribution	1-way ANOVA, Bonferroni post hoc	F(2, 15)=257.8	P<0.0001<br>Sham vs ICH p<0.0001<br>ICH vs ICH+P7C3-A20 p<0.0001
SF3C	Sham=6<br>ICH=6<br>ICH+P7C3-A20=6	Normal distribution	1-way ANOVA, Bonferroni post hoc	F(2, 15)=8.703	P=0.0031<br>Sham vs ICH p=0.0040<br>ICH vs ICH+P7C3-A20 p=0.0186
SF3D	Sham=6<br>ICH=6<br>ICH+P7C3-A20=6	Normal distribution	1-way ANOVA, Bonferroni post hoc	F(2, 15)=30.34	P<0.0001<br>Sham vs ICH p<0.0001<br>ICH vs ICH+P7C3-A20 p<0.0001
Figure S4	n	Data structure	Test used	Statistic	P value
SF4B	Vehicle=6<br>ICH=6<br>ICH+P7C3-A20=6<br>CCCP=6	Normal distribution	1-way ANOVA, Bonferroni post hoc	F(3, 20)=27.12	P<0.0001<br>Vehicle vs ICH p=0.0002<br>ICH vs ICH+P7C3-A20 p=0.2392
SF4C	Vehicle=6<br>ICH=6<br>ICH+P7C3-A20=6<br>CCCP=6	Normal distribution	1-way ANOVA, Bonferroni post hoc	F(3, 20)=83.61	P<0.0001<br>Vehicle vs ICH p<0.0001<br>ICH vs ICH+P7C3-A20 p=0.9555
SF4D	Vehicle=6<br>ICH=6<br>ICH+P7C3-A20=6<br>CCCP=6	Normal distribution	1-way ANOVA, Bonferroni post hoc	F(3, 20)=40.17	P<0.0001<br>Vehicle vs ICH p<0.0001<br>ICH vs ICH+P7C3-A20 p>0.9999
