# Supplementary figures and images for: P7C3-A20 Attenuates Microglial Inflammation and Brain Injury after ICH through Activating the NAD+/Sirt3 Pathway
Source: Oxid Med Cell Longev. 2023 Feb 8;2023:7857760. doi: 10.1155/2023/7857760 (PMC9936507; doi:10.1155/2023/7857760)

Supplementary Fig. 1

GO Enrichment BarPlot

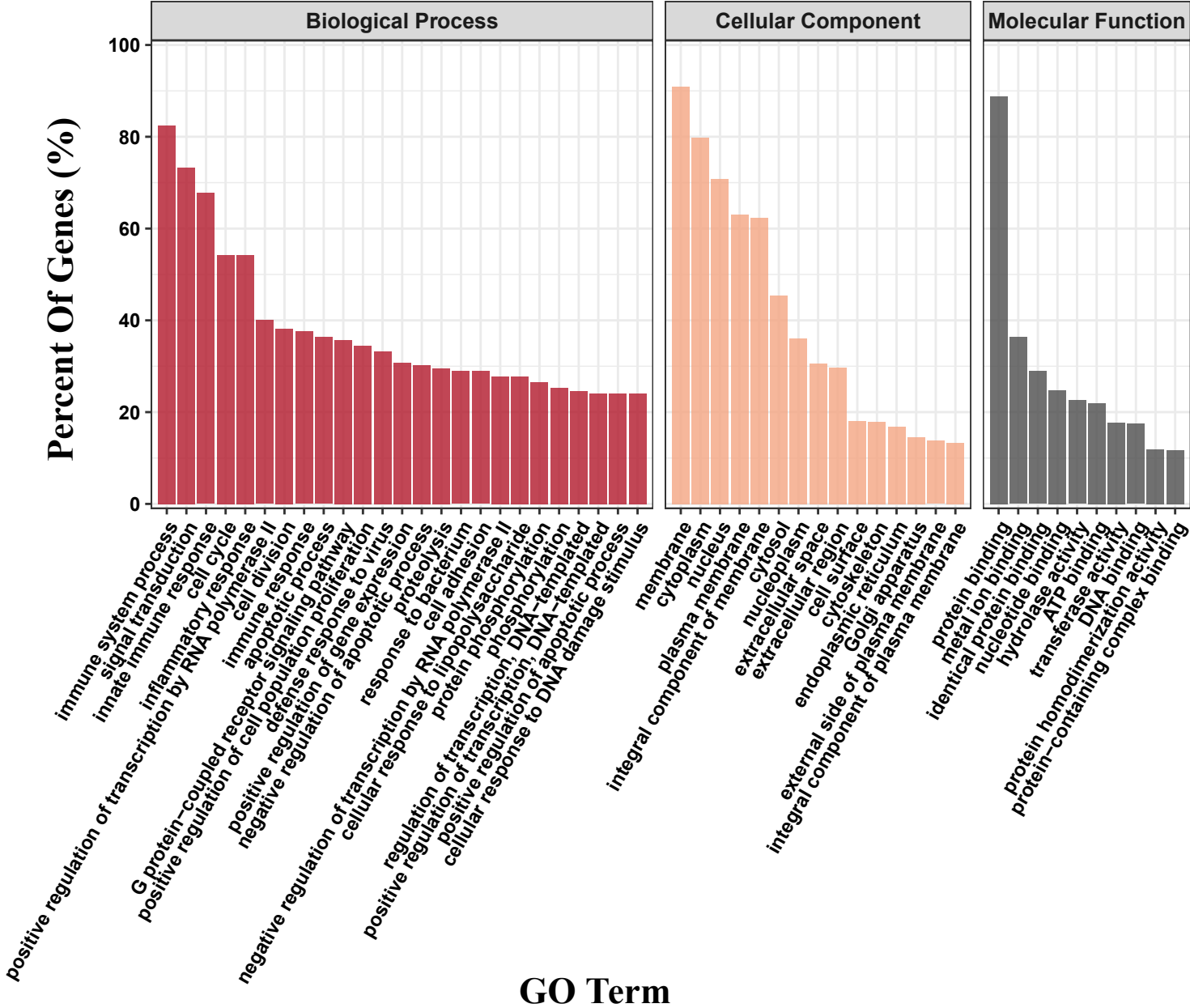

Supplement: Supplementary 3 — Supplementary Figure 1: GO enrichment analysis of the overlapped genes in the Venn diagram. [file 7857760.f3.pdf]

Supplementary Fig. 2

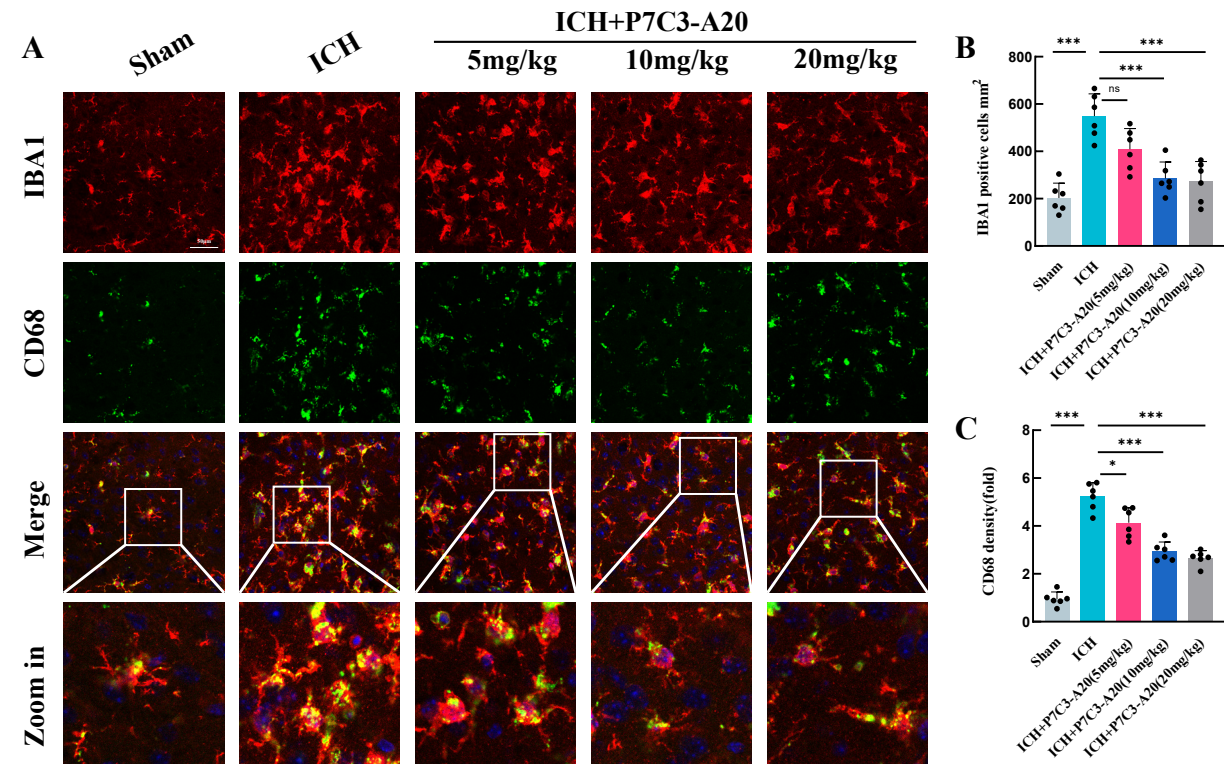

Supplement: Supplementary 4 — Supplementary Figure 2: the neuroprotective effects of the different doses of P7C3-A20 on neuroinflammatory injury after ICH. (A) Immunofluorescence staining for Iba1 (red) with CD68 (green) revealing the activated microglia levels in different dose groups. Each micrograph next to the boxed area shows an amplified version of the white square in merged images. Scale bar, 50 μm. (B) Statistical analysis of activated microglia number. ∗∗∗P < 0.001, ns. P > 0.05, and n = 6. (c) Analysis of the quantitative fluorescence intensity for CD68. ∗P < 0.05, ∗∗∗P < 0.001, and n = 6. Values are expressed as mean ± SD. Significance was determined by one-way ANOVA with Bonferroni post hoc tests (B, C). [file 7857760.f4.pdf]

**Supplementary Fig. 3**

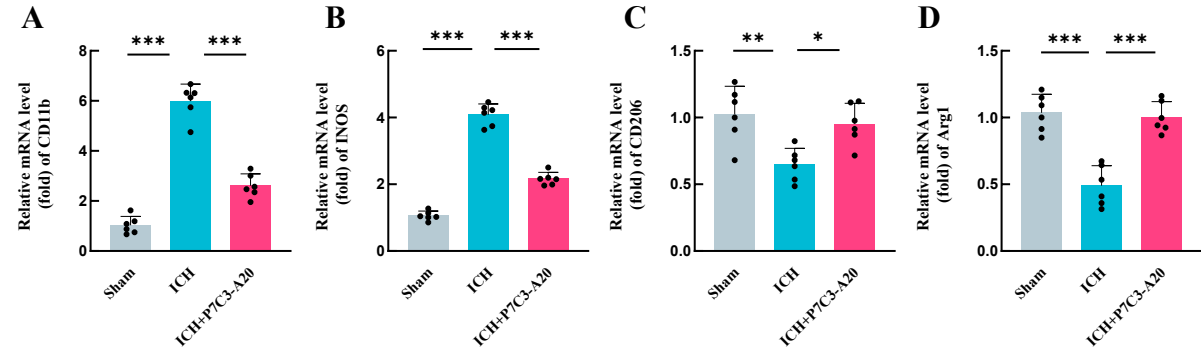

Supplement: Supplementary 5 — Supplementary Figure 3: qPCR was used to validate the trend in the expression of microglial activation marker at the transcriptional level. (A) CD11b mRNA level. ∗∗∗P < 0.001 and n = 6. (B) IONS mRNA level. ∗∗∗P < 0.001 and n = 6. (C) CD206 mRNA level. ∗P < 0.05, ∗∗P < 0.01, and n = 6. (D) Arg1 mRNA level. ∗∗∗P < 0.001 and n = 6. As shown in A-D, the ICH group markedly increased CD11b and INOS and decreased CD206 and Arg1 at the transcriptional level compared to the sham, while those trends were reversed by additional P7C3-A20 treatment. Values are expressed as mean ± SD. Significance was determined by one-way ANOVA with Bonferroni post hoc tests (A, B, C, and D). [file 7857760.f5.pdf]

**Supplementary Fig. 4**

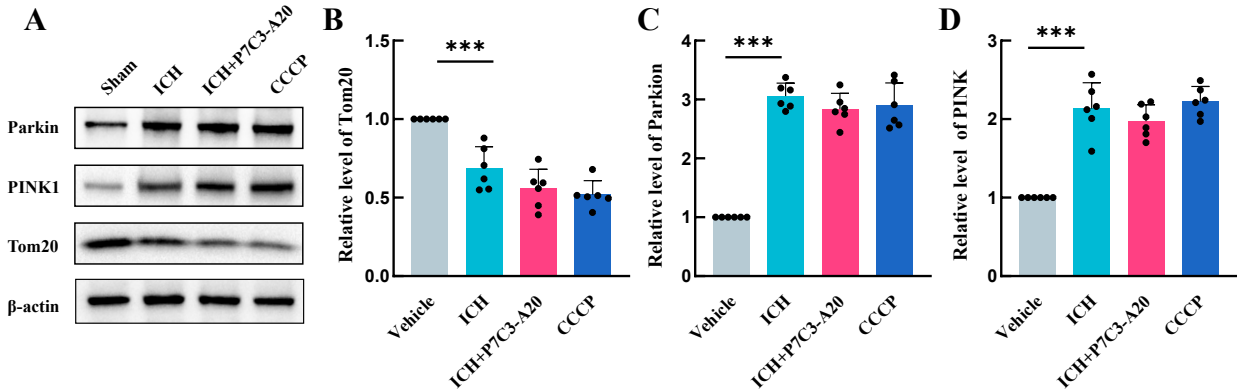

Supplement: Supplementary 6 — Supplementary Figure 4: P7C3-A20 showed no effect on the OxyHb-induced mitochondrial autophagy process. (A-D) Representative Western blot images of Parkin, PINK1, and Tom20 and the related statistical analysis. Western blotting displayed an evident fall of Tom20 and a rise of Parkin and PINK1 in the ICH group compared to the vehicle, while those changes were not reversed by additional P7C3-A20 treatment. Notably, there was no statistical significance between ICH, ICH+P7C3-A20, and CCCP (positive control) groups. ∗∗∗P < 0.001, ICH vs. sham. Values are expressed as mean ± SD. Significance was determined by one-way ANOVA with Bonferroni post hoc tests (B, C, and D). [file 7857760.f6.pdf]
